# Novel Carbazole-Piperazine Hybrid Small Molecule Induces Apoptosis by Targeting BCL-2 and Inhibits Tumor Progression in Lung Adenocarcinoma In Vitro and Xenograft Mice Model

**DOI:** 10.3390/cancers11091245

**Published:** 2019-08-25

**Authors:** Raj Kumar Mongre, Chandra Bhushan Mishra, Amresh Prakash, Samil Jung, Beom Suk Lee, Shikha Kumari, Jin Tae Hong, Myeong-Sok Lee

**Affiliations:** 1 Molecular Cancer Biology Laboratory, Cellular Heterogeneity Research Center, Department of Biosystem, Sookmyung Women’s University, Hyochangwon gil-52, Yongsan-Gu, Seoul 140-742, Korea; 2 Dr. B.R. Ambedkar Center for Biomedical Research, University of Delhi, Delhi 110007, India; 3 Amity Institute of Integrative Sciences and Health (AIISH), Amity University Haryana, Amity Education Valley, Gurgaon 122413, India; 4 College of Pharmacy and Medical Research Center, Chungbuk National University, Cheongju 28160, Korea

**Keywords:** carbazole-piperazine hybrid molecule, ECPU-0001, tumor xenograft, mitochondrial mediated apoptosis, intrinsic pathway, molecular dynamics simulation

## Abstract

Lung cancer is a type of deadly cancer and a leading cause of cancer associated death worldwide. BCL-2 protein is considered as an imperative target for the treatment of cancer due to their significant involvement in cell survival and death. A carbazole-piperazine hybrid molecule ECPU-0001 was designed and synthesized as a potent BCL-2 targeting agent with effective anticancer cancer activity. Interaction of ECPU-001 has been assessed by docking, molecular dynamics (MD) simulation, and thermal shift assay. Further, in vitro and in vivo anticancer activity was executed by cytotoxicity assay, FACS, colony formation and migration assay, western blotting, immunocyto/histochemistry and xenograft nude mice model. Molecular docking and MD simulation study confirmed that ECPU-0001 nicely interacts with the active site of BCL-2 by displaying a Ki value of 5.72 µM and binding energy (ΔG) of –8.35 kcal/mol. Thermal shift assay also validated strong interaction of this compound with BCL-2. ECPU-0001 effectively exerted a cytotoxic effect against lung adenocarnoma cells A459 with an IC_50_ value of 1.779 µM. Molecular mechanism of action have also been investigated and found that ECPU-0001 induced apoptosis in A459 cell by targeting BCL-2 to induce intrinsic pathway of apoptosis. Administration of ECPU-0001 significantly inhibited progression of tumor in a xenograft model without exerting severe toxicity and remarkably reduced tumor volume as well as tumor burden in treated animals. Our investigation bestowed ECPU-0001 as an effective tumoricidal agent which exhibited impressive anticancer activity in vitro as well as in vivo by targeting BCL-2 associated intrinsic pathway of apoptosis. Thus, ECPU-0001 may provide a valuable input for therapy of lung adenosarcoma in future, however, further extensive investigation of this compound will be needed.

## 1. Introduction

Lung cancer is considered as one of the most prevalent malignant diseases and foremost cause of cancer death worldwide [1,2]. WHO has classified lung cancer in two categories, small cell lung cancer (SCLC) and non-small cell lung cancer (NSCLC) [3,4]. It is estimated that more than 1.3 million new cases of lung cancer each year are appearing. The majority of lung cancer is not curable at the time of diagnosis and displays a remarkable insensitiveness to chemotherapy and radiation therapy [5]. Over 85% of these patients eventually die from disseminating disease during the first 5 years and this extreme mortality has not changed significantly during the last three decades [6]. Current treatment strategies of lung cancer include chemotherapy, radiotherapy, immunotherapy and surgical removal of uncontrolled malignant cells [7]. In the past decades, widespread efforts have been made to fight with malignancies and due course several anticancer drug molecules have been developed [8]. However, currently running anticancer drugs in market is suffering from numerous drawback including normal cell toxicity, resistance and intolerance. Thus, the discovery of powerful anticancer agents is a compulsory requirement to selectively kill cancerous cell without exerting severe side effect.

Apoptosis is a vital process to maintain homeostatic balance between the rates of cell growth and programmed-cell-death, which is necessary to sustaining normal physiological phenomenon [9,10]. Impairment in natural apoptosis mechanisms play crucial role in tumor progression, permitting neoplastic cell survival and offering protection from oxidative stress which lead in uncontrolled growth of cancer cells [11]. Therefore, activation of apoptosis in cancer cells considered as key target to stop cancer progression and numerous anticancer drug followed this strategy to halt tumor’s growth speed. The mitochondrial mediated apoptotic pathway is established as the key pathway in anticancer drug-mediated cell killing [11,12]. The mitochondria play a vital role in apoptotic pathway and control several death effector signals by discharge of apoptotic proteins such as the APAF-1 and Cytochrome-C [13]. BCL-2 family proteins are considered as a main regulator of the mitochondrial apoptotic pathway by upregulating BAK and BAX [14]. Indeed, BCL-2 emerged as a valuable target to stimulate apoptosis in various cancer cells [15]. In the past decade, several potent small molecule as BCL-2 inhibitors have been developed which demonstrated effective anti-cancer activity in vitro as well as in vivo. For example, small molecules ABT-737, Navitoclax and Venetoclax are potent BCL-2 inhibitors, which have shown promising efficacy against cancers including lung cancer [16,17,18]. Nevertheless, these BCL-2 inhibitors are suffering with several drawbacks such as low water solubility and severe unwanted side effects including thrombocytopenia [16]. Therefore, the discovery of potent and safe BCL-2 targeting small molecules is necessarily required to impede growth of cancer cells.

The molecular hybridization of biologically active molecules is appeared to be a powerful tool for generating highly effective drug molecules, especially against complex disease like cancer [19]. Hybrid drug molecules may serve better than combinational therapy approaches in which cocktails of drugs are the main choice [20]. Drug cocktails raise several challenges such as the extent of pharmacokinetics, drug interactions, metabolism and bioavailability. Therefore, a hybrid molecule, which consists of the assimilation of two or more pharmacophores into a single molecule, has been got a great attention in modern drug discovery approach. This strategy bestowed a number of potential drug molecule which has shown excellent efficacy in clinical and pre-clinical studies. For example, Estramustine, a hybrid anticancer drug molecule running in the market has shown excellent efficacy clinically [21].

Carbazole is an important heterocyclic compound with tricyclic structure and widely used to discover potent anticancer agents [22,23]. Numerous natural and synthetic carbazoles such as clausine-B, ellipticin, mahanimbine, mukonine, rebaccamycin, and heptaphyline have been known for their excellent antineoplastic activity [23,24,25,26,27,28]. Additionally, various carbazole derivatives have been also synthesized by several research groups which displayed admirable anticancer activity in a wide range of in vitro as well as in vivo models [23].

On the other hand, the piperazine moiety has also received significant attention to discover numerous potent biologically active molecules, including anti-proliferative agents [29,30]. Piperazine containing anticancer drug Imatinib is successfully running in the market and used to treat several types of cancer, including myelogenous leukemia [31]. Another piperazine bearing anticancer drug Palbociclib has been recently approved by FDA for the treatment of metastatic breast cancer [32]. Additionally, ARIAD Pharmaceuticals have also developed piperazine consisting anticancer drug Ponatinib for the therapy of chronic myeloid leukemia [33].

Besides these, the urea functional group has been also validated to generate potent anticancer activity and installation of this group enhanced the anticancer effect in various reports [34]. Leggans et al. have investigated that insertion of ethyl urea at the C20′ of known anticancer drug vinblastine significantly enhance its potency and showed excellent cytotoxicity in vinblastine-resistant tumor cell line [35,36].

**Figure 1:**
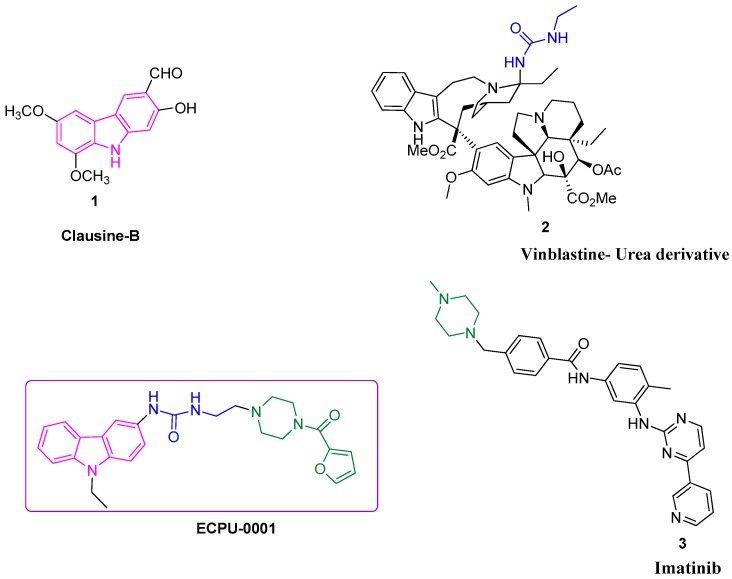
Potent anticancer molecules in preclinical or clinical trial studies and designed novel hybrid small molecule ECPU-0001.

Considering these facts, we have hybridized carbazole and piperazine pharmacophore using ethyl urea linker to discover potent BCL-2 targeting anti-cancer agent ECPU-0001 (Figure 1). It is assumed that hybridization of these pharmacologically active pharmacophores through anticancer generating linker ethyl urea will provide a potent molecule which may endow superior anticancer activity. The best of our knowledge, there is no reports available regarding such type of hybrid molecule, therefore, this study may provide a novel and potent anticancer agent for management of lung cancer.

Designed molecule was initially assessed by using in-silico tools such as docking as well as molecular dynamic (MD) study to confirm its interaction with BCL-2. After getting promising output from in-silico study, ECPU-0001 was synthesized and extensively evaluated for their affinity with BCL-2 and anticancer activity in vitro as well as in vivo. Our in-silico and in vitro investigation revealed that EPU-0001 effectively target BCL-2 protein to induce apoptosis in cancer cells. ECPU-0001 has been evaluated for their cytotoxicity ability against NCIH-1299, U2OS, A549, HT-29, MCF7 and Hela. The result indicates that ECPU-0001 showed excellent cytotoxicity against lung adenosarcoma A549 cells with an IC_50_ value of 1.77 µM. Mechanistic investigation revealed that ECPU-0001 induces apoptosis through mitochondrial mediated intrinsic apoptotic pathway by targeting BCL-2 and arrested the cell cycle by inhibiting CDK6 as well as cyclin D1. ECPU-0001 also endowed with excellent in vivo efficacy and significantly reduced tumor progression in nude mice model. Hence, present investigation grants a foundation for development of ECPU-0001 as novel and potent anticancer agents which may play a crucial role in lung adenosarcoma treatment in future.

## 2. Results

### 2.1. Molecular Interaction of ECPU-0001 with BCL-2: Molecular Docking and MD Simulation Study

**Figure 2:**
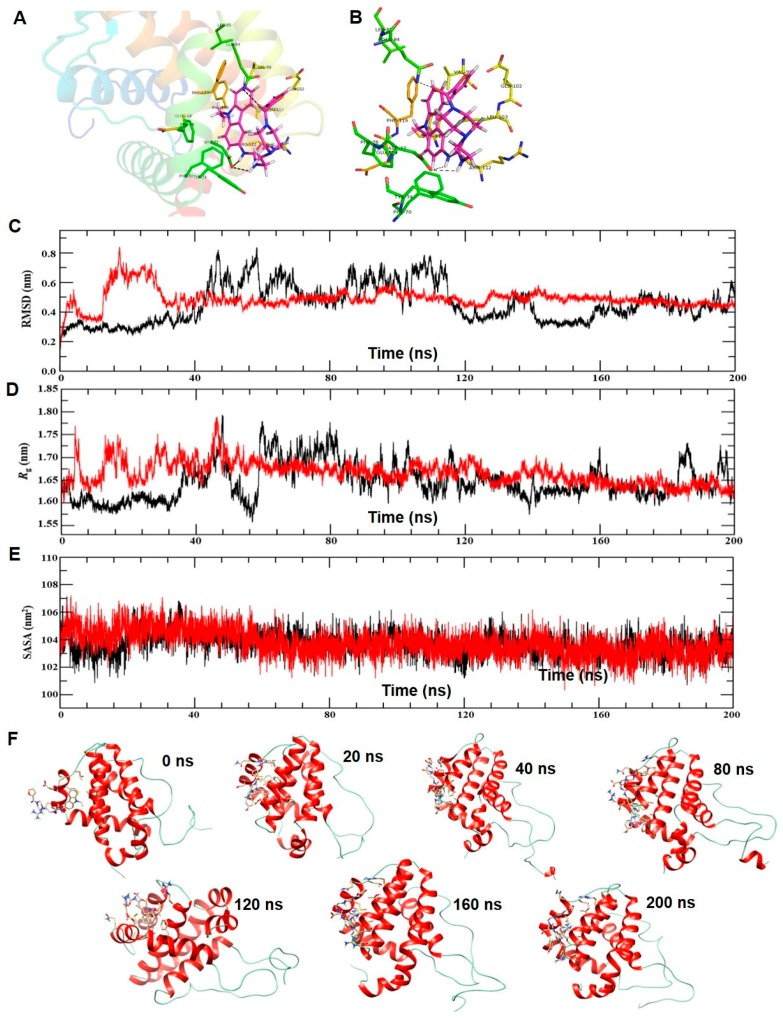
BCL-2-ECPU-0001 interaction by molecular docking and molecular dynamics (MD) simulations. (**A**) Stereo view of BCL-2-ligand complex at PyMol. The cartoon view of BCL-2 docked with ligand. The active site residues and ligand are represented with stick. (**B**) Detail view of interatomic interactions of active site residues and ligand. The active sites residues are shown with stick and the ligand is represented with ball and stick model. (**C**) Time evolution plot of root mean square deviation (RMSD) plot of BCL-2 (black) and BCL-2-ligand complex (Red) in water at 300 K during the MD simulation of 0–200 ns. (**D**) Time evolution plot of radius of gyration (R_g_) plot of BCL-2 (black) and BCL-2-ligand complex (Red). (**E**) Time evolution plot of SASA. Protein BCL-2 shown with color black and BCL-2-ligand complex is represented with color red. (**F**) Structural snap of BCL-2-ligand at the different time interval of MD simulation in water at 300 K. The ligand and active site residues involve in interactions are shown with stick. The cartoon view of BCL-2-ligand at molecular visualizer tool Chimera 1.13.

To understand the spatial binding of ECPU-001 in the active site of BCL-2, a molecular docking study has been performed using AutoDock 4.2. We adopted the recently solved crystal structure of BCL-2 complex with inhibitor S55746 which is suggested that active site of BCL-2 is canonically formed by four α-helices (α1–α4) and the inhibitor is transversely occupied in the active site, stabilized with hydrophobic residues of α2 and α4 [37]. The molecular docking result shows that ECPU-0001 is apparently occupying the same preferential spatial orientation in the active site of BCL-2 and involved in H-bond interaction with the active site residues Asp77 and Gln84 (Figure 2A). The urea moiety of ECPU-001 bridging, carbazole and propylpiperazine having poplar interaction at active site. Furan ring the carbazole moiety surrounded with hydrophobic residues of α2, α3 and α4, respectively. However, carbazole moiety is deeply orientated into the active site and stabilized by hydrophobic interactions with Phe70, Tyr74, Phe78, Phe78, Phe119 and Phe116 (Figure 2A,B), suggesting that ECPU-0001 is strongly interacted with active site amino acids, displayed a Ki value of 5.72 µM with a binding energy (ΔG) of –8.35 kcal/mol. Furthermore, a MD simulation study has been also executed to investigate the conformational stability of ECPU-0001 in active site of BCL-2, in water at 300 K. To examine the structural stability of BCL-2 and BCL-2-ECPU-0001 complex, we monitored the time evolution plot of all C^α^-atom RMSD as shown in Figure 2C. In this figure, we can see that the RMSD trajectory of BCL-2 in water remains stable during 0–40 ns. However, the continuous drifts of ~0.25 is propagated ~40–120 ns which dropdown around ~120 ns and the stable structure can be seen during the last 80 ns of simulation. Whereas, RMSD trajectory of BCL-2-ECPU-0001 complex shows drifts of ~0.25 nm during the initial 15–30 ns of simulation which is settled ~32 ns and a stable equilibrium can be seen till the simulation finished at 200 ns (Figure 2C).

Radius of gyration (*R_g_*) is another effective parameter to examine the structural integrity of protein. The time evolution plot of *R_g_* also shows stable trajectory of BCL-2 at initial 0–35 ns followed by consecutive drifts during 40–120 ns (Figure 2D). The *R_g_* trajectory achieves the stable equilibrium around 120 ns and remains stable till the simulation end at 200 ns. We can observe quite stable trajectory of *R_g_* obtained for BCL-2-ligand complex, during the initial perturbation in structure which is obvious due to protein-ligand interaction, the trajectory remains stable around the average change in *R_g_* value 1.70 nm.

In addition, we also examine the solvent accessible surface area (SASA) to ensure the structural stability of BCL-2-ECPU-0001 complex. The time evolution plot of SASA shows marginal change in structure of BCL-2 during the initial 15–30 ns and the remaining period of simulation trajectory remains stable and completely overlapped with the SASA trajectory of BCL-2-ECPU-0001 complex (Figure 2E). The trajectory of both, protein and protein-ligand complex converged around 40 ns having average SASA value 104 nm^2^ which provide the clear evidence of stable interaction of ligand with BCL-2. Furthermore, we monitored the RMSF plots which is representing the local changes in the structure of BCL-2 on the binding of ligand. The result shows the reduction in the average fluctuation of residues belonging to N and C-terminal helices, α-helices-2, 3 and 4 and loops connecting then α-helices-3 and 4, and α-helices-4 and 5 which shows the stable spatial interaction of ligand in the active site of BCL-2 (Figure S1). However, we find the marginal change in secondary structure, plot suggests that binding of ligand does not alter the secondary conformation of BCL-2 and spatially remains fitted at the active site during the simulation of 200 ns (Figures S2 and S3). Thus, to provide the elegance view of BCL-2-ligand interaction, we also captured the structural snapshot of complex at the time interval of 0, 20, 40, 80, 120, 160 and 200 ns as shown in Figure 2F. Result shows that the ligand is better fitted into the active site of BCL-2 and stabilized with intermolecular interaction which remained persistent for the longer period of 0–200 ns.

### 2.2. Synthesis of Novel Carbazole-Piperazine Hybrid Molecule (ECPU-0001)

**Scheme 1:**
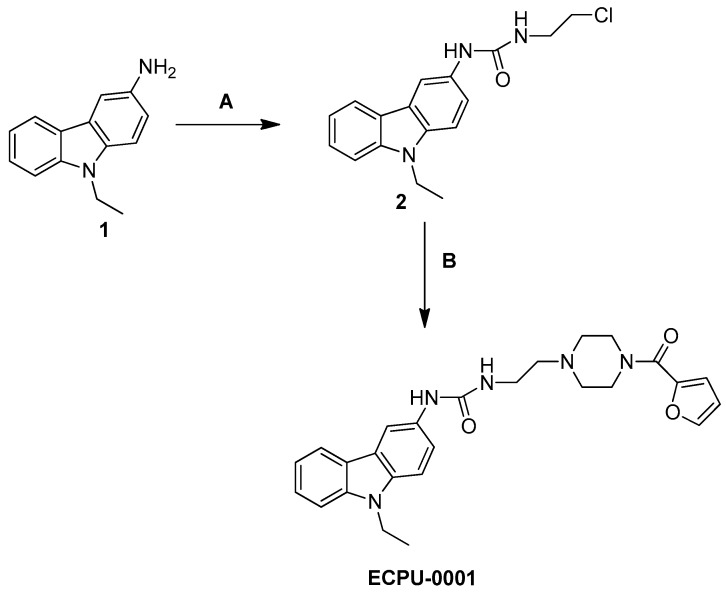
Reagents & conditions. **A**: 2-Chloroethyl isocyanate, THF, 0–5 °C, 12 h, **B**: 2-furoyl piperazine, NaI, NaHCO_3_, THF, reflux, 14 h.

**Figure 3:**
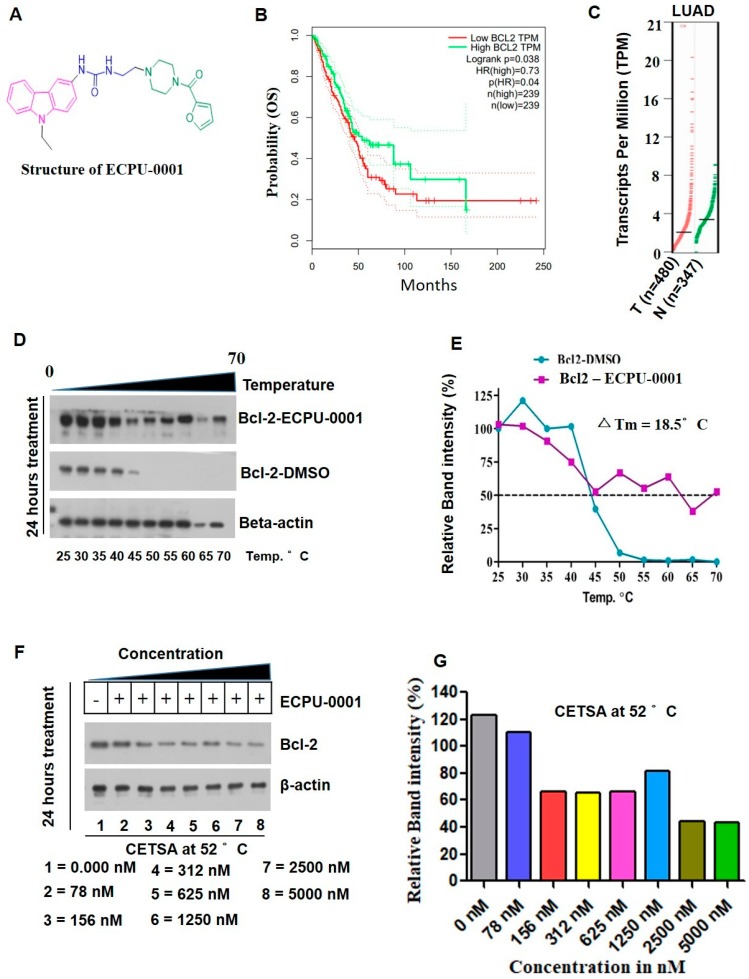
ECPU-0001 strongly interacts with BCL-2 in vitro. (**A**) Structure of novel hybrid molecule ECPU-0001. (**B**) Kaplan Meier survival curve showed overexpression of BCL-2 affect patient’s survival in adeno carcinoma of lung patients. Patient’s survival analyzed and obtained from GEPIA online web portal. (**C**) Scatter plot showed an elevated mRNA levels of BCL-2 in adenolung cancer patient’s samples compared with normal lung tissue which is derived from GEPIA analysis. (**D**) The cellular thermal shift assay (CETSA) was performed to evaluate the engagement and interaction between ECPU-0001 and BCL-2 in cell lysates (in vitro). After incubation with a compound ECPU-0001 (2.0 μM) or a vehicle control (DMSO), cells were subjected to aliquot then heated in ten different temperatures (from 25 °C to 70 °C). Subsequently, an aggregated proteins precipitation is spin downed by centrifugation at 3000 rpm speed for 2 min. The rest of soluble protein lysate is examined by immunoblotting. (**E**) Relative band intensity percentage was calculated using ImageJ software and determined ΔTm value. (**F**) Further, to determine the inhibitory dose of ECPU-0001 to reduce the translation of BCL-2, 2 μM of ECPU-0001 treated to lung cancer cells and then performed isothermal cellular engagement assay using Isothermal CETSA. (**G**) BCL-2 protein relative expression in terms of band intensity was calculated using ImageJ software.

Synthesis of designed hybrid molecule ECPU-0001 was carried in three step process (Scheme 1). In brief, commercial accessible carbazole amine 1 was reacted with chloroethyl isocyanate in anhydrous tetrahydrofuran (THF) to achieve chloroethyl urea derivative 2. Further, chloroethyl urea derivative 2, and 2-furoyl piperazine were refluxed in THF by adding catalytic amount of NaHCO_3_ and NaI to accomplish target compound 1-(9-Ethyl-9H-carbazol-3-yl)-3-{2-[4-(furan-2-carbonyl)-piperazin-1-yl]-ethyl}-urea (ECPU-0001) (Figure 3A). The synthesized hybrid molecule has been characterized by ^1^H NMR, ^13^C NMR and Mass spectroscopy. The purity of this molecule has been analyzed by Reverse phase HPLC.

### 2.3. ECPU-0001 Appeared as a Strong BCL-2 Targeting Agent In Vitro Study

BCL-2 actively associated with the most of tumor induction and an elevated levels of BCL-2 significantly reduced the survival of lung cancer patients (Figure 3B,C). To confirm engagement of ECPU-0001 towards BCL-2 in the cells, we performed cellular thermal shift assay (CETSA) followed the previous study [38]. When subjected to heat denaturing of cellular BCL-2 protein, lost their higher order structure, unfold and denatured at around 50–52 °C in the absence of ECPU-0001 (DMSO treated group). On the other hand, in the exposure of ECPU-0001 at 2.0 μM concentration, but not of the control protein β-actin, were enhanced stability even in 65–70 °C (Figure 3D,E). ECPU-0001 showed temperature dependent thermal shift, with a ΔTm values of 18.5 °C, while the DMSO treatment group had no such thermal stabilization effect on BCL-2 protein. Further, the binding of ECPU-0001 with BCL-2 was also verified by investigating target interaction potency of ECPU-0001. For this investigation, we conducted CETSA analysis on concentration dependent manner. ECPU-0001 efficiently stabilized BCL-2 protein in the concentration range of 78 mm to 5.0 μM compared to DMSO treatment (Figure 3F). This thermal stability of the BCL-2 protein was reduced up to 50% at 2.23 μM concentration of ECPU-0001 (Figure 3G). These data strongly suggest that ECPU-0001 is efficiently targeting the BCL-2 to induce apoptosis in A549 Cells. Our western blot analysis also supports inhibition of BCL-2 by CEPU-0001, where BAX protein is significantly up-regulated after treatment of ECPU-0001.

### 2.4. Cytotoxic Potential of ECPU-0001 in Cancer Cell Lines

**Figure 4:**
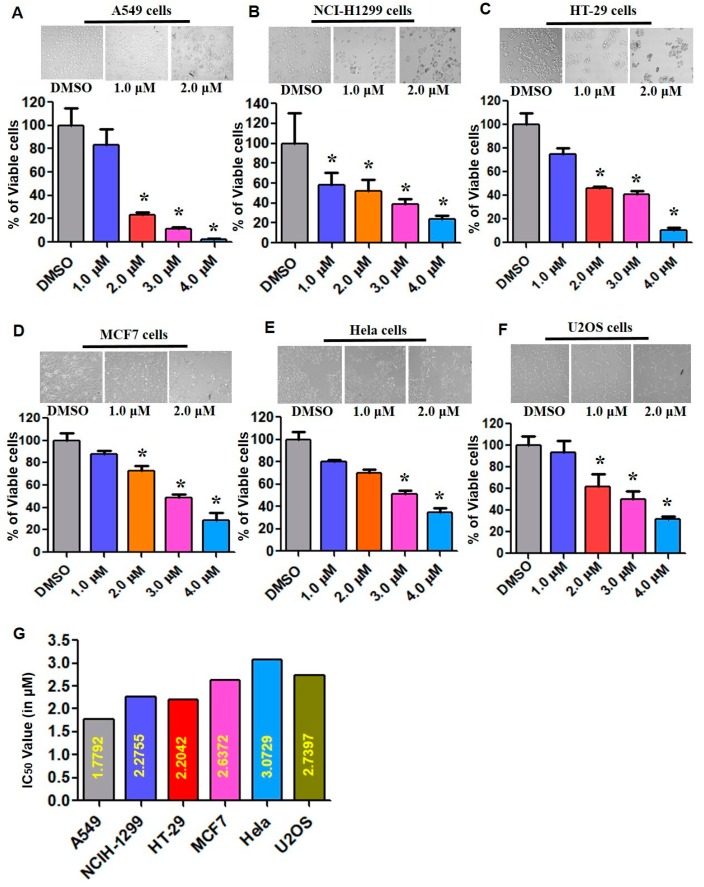
Tumoricidal activities of ECPU-0001 in numerous cell lines and colony forming inhibition. (**A**) Cellular cytotoxicity assay performed in A549. (**B**) NCI-H1299. (**C**) HT-29 (**D**) MCF7, (**E**) Hela, and (**F**) U2OS cancer cell lines. Approximately 0.5 × 10^6^ cells/well were seeded in 60 mm cell culture plates and treated with ECPU-0001 1.0, 2.0, 3.0, and 4.0 µM/mL for 24 h; 0.2% dimethylsulfoxide (DMSO) in medium was used as a control. (**G**) IC_50_ values for different types of cancer cell lines using the cell cytotoxicity assay. Magnification Scale bar: 20×. * *p* < 0.05.

In vitro cytotoxicity potential of ECPU-0001 was evaluated against six cancer cell lines; NCIH-1299, U2OS, A549, MCF7, HT-29 and Hela by Trypan-Blue EVE™ Automatic Cell Counter assay. The result showed that ECPU-0001 displayed admirable to excellent cytotoxicity against six cell lines (Figure 4A–F). ECPU-0001 have shown IC_50_ values of 1.779, 2.270, 2.20, 2.637, 3.072 and 2.739 µM against A549, NCI-H1299, HT-29, MCF7, Hela, and U2OS cells respectively (Figure 4G). Appreciably, ECPU-0001 exerted an excellent cytotoxic effect (IC_50_ = 1.779 µM) against A549 as compared to other tested cell lines (Figure 4G). Phase contrast microscopy study (morphological analysis) also validated the cytotoxicity potential of ECPU-0001 against A549 cells, where significant cell death was observed with treatment of ECPU-0001.

### 2.5. ECPU-0001 Impedes Cancer Growth and Migration In Vitro

**Figure 5:**
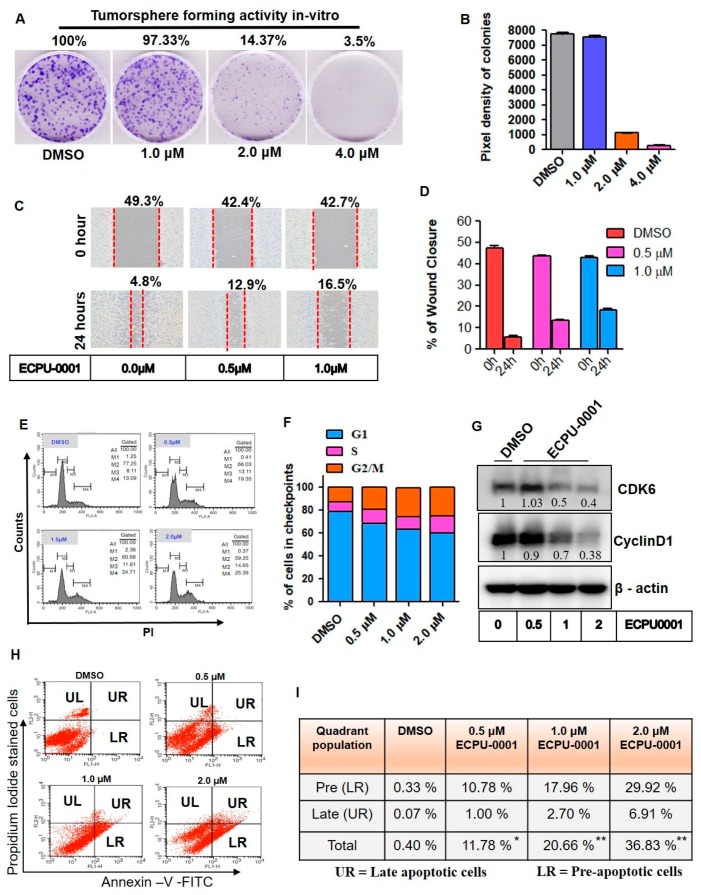
ECPU-0001 inhibits cancer growth, migration via CyclinD1/CDK6 mediated cell cycle G2/M phase arrest. (**A**) Colony formation assay was done to investigate long-term effect of ECPU-0001 on proliferation of lung cancer tumorigenic cells. Data are means ± SD of three independent experiments. Opted Scale bar: 20× (**B**) Tumorspheres or colonies were analyzed and pixel density was determined using ImageJ software. (**C**) To elucidate the effect of ECPU-0001 on A549 cell invasion and migration, briefly cells were propagated in 6-well plates until it covered near to 80% confluency. Then, each well scratched with 200 μL tip equally under the same condition. The wounded A549 cells-layered cultured and incubated in without FBS RPMI-1640 medium. Then cells were treated medium which containing 0, 0.5, and 1.0 μM of ECPU-0001 for 24 h. Scale bar: 20× (**D**) Percentage of wound healed area was measured using ImageJ followed standard protocol. (**E**,**F**) The cell cycle sub-phase in different checkpoints was analyzed using FACS. A549 cells were treated with vehicle (DMSO; control), 0.5 μM, 1.0 μM and 2.0 μM ECPU-0001 for 24 h, followed by FACS protocol. In the histogram peak, the horizontal line depicts the intensity of propidium iodide that is content of DNA while vertical line reveals total cell number. (**G**) Immunoblot analysis of cell cycle regulator proteins. (**H**) ECPU-0001 induces pre/late apoptosis and in-cell engagement of BCL-2. ECPU-0001 accelerate lung cancer cell apoptosis through enhancing the activity of Annexin-V-FITC/PI positive cells. Apoptosis was tested by FACS analysis after A549 cells were treated with ECPU-0001 for 24 h. (**I**) The apoptotic (pre and late apoptotic cells) percentage was evaluated and shown in table.

Next, the tumor sphere formation assay was employed to study the inhibitory effect of hybrid molecule ECPU-0001 on the A549 cells proliferation. In this study, A549 cells were propagated with or without various concentrations like 1.0, 2.0 and 4.0 μM ECPU-0001 for 6 days and the number and diameter of tumor spheres were quantified using ImageJ software. The result showed that even 1.0 and 2.0 µM concentrations of ECPU-0001 significantly inhibited the colony formation capacity in this cell line as shown in Figure 5A,B. The, result in Figure 5B showed that ECPU-0001 greatly suppressed not only the formation of tumor spheres also reduced its efficiency. Remarkably, treatment with 2.0 μM resulted in approximately 14.37% reduction in clonogenicity and tumor sphere formation, whereas higher concentration (4.0 μM) were more inhibitory (Figure 5A). Further, the effects of ECPU-0001 on cell migration and invasion were also studied by treating ECPU-0001 to A549 cells for 24 h and the migrated cells area have been determined through ImageJ software. The result of this study indicated that exposure of ECPU-0001 significantly depletes the migration and invasion ability of A549 adenolung cancer cells (Figure 5C,D). Additionally, ECPU-0001 significantly inhibited A549 cell migration concentration dependently as compared to control. ECPU-0001 treatment at the concentration 0.5 µM and 1.0 µM displayed 12.9% and 16.5% remaining wound as compared to control which has displayed 4.8% remaining wound after 24 h. Thus, ECPU-0001 successfully able to reduce lung cancer cells growth and migration in a concentration dependent manner.

### 2.6. ECPU-0001 Augments CyclinD1/CDK6 Mediated Cell Cycle Arrest and Apoptosis

Tumor cell growth is the result of dysregulation of cell divisions; therefore, inhibition of cell cycle has been validated as an imperative target for the treatment of cancer. To confirm the cell cycle arresting potential of ECPU-0001, a flow-cytometric analysis was performed at 0.5, 1.0 and 2.0 µM concentrations of ECPU-0001. The result indicated that ECPU-0001 effectively arrested G2/M phase of cell cycle and significantly enhanced A549 cell population concentration dependently as compared to control. ECPU-0001 treatment showed 19.35, 24.71 and 25.39% cell population in G2/M phase at 0.5, 1.0 and 2 µM concentration, respectively, whereas untreated cells displayed 13.09% cells in this phase (Figure 5E,F). Further, to elucidate molecular target of ECPU-0001 associated with cell cycle arrestation, a western blot analysis was performed. Western blot analysis indicated that ECPU-0001 significantly reduced expression of cell cycle regulatory protein Cyclin D1 and kinase CDK6. (Figure 5G and Figure S4). Hence, ECPU-0001 forcefully inhibited cell cycle progression in A549 cells by targeting Cyclin D1 as well as CDK6.

Further, we also checked apoptotic potential of ECPU-0001 in A549 cells by double staining with propidium iodide and Annexin-V-FITC. In contrast, flow-cytometry analysis of double stained cells showed four types of sub-population of cells, namely live cells (Annexin-V^negative^/PI^negative^), early apoptotic cells (Annexin-V^positive^ PI^negative^), late apoptotic cells (Annexin-V^positive^ PI^positive^) and necrotic cells (Annexin-V^negative^ PI^positive^) cells. As shown in Figure 5H,I, ECPU-0001 treatment at 0.5, 1.0 and 2.0 µM concentrations resulted in a higher sub-population of early apoptosis 10.78%, 17.96% and 29.92% respectively in A549 cells compared to control (0.33%). Similarly, there were dose-dependent significant enhancement of late apoptotic sub-population from 0.07% to 16.91% when treated with 0.5, 1.0 and 2.0 µM of ECPU-0001.

### 2.7. ECPU-0001 Induces Apoptosis through the Mitochondria Mediated Intrinsic Pathway by Targeting BCL-2

**Figure 6:**
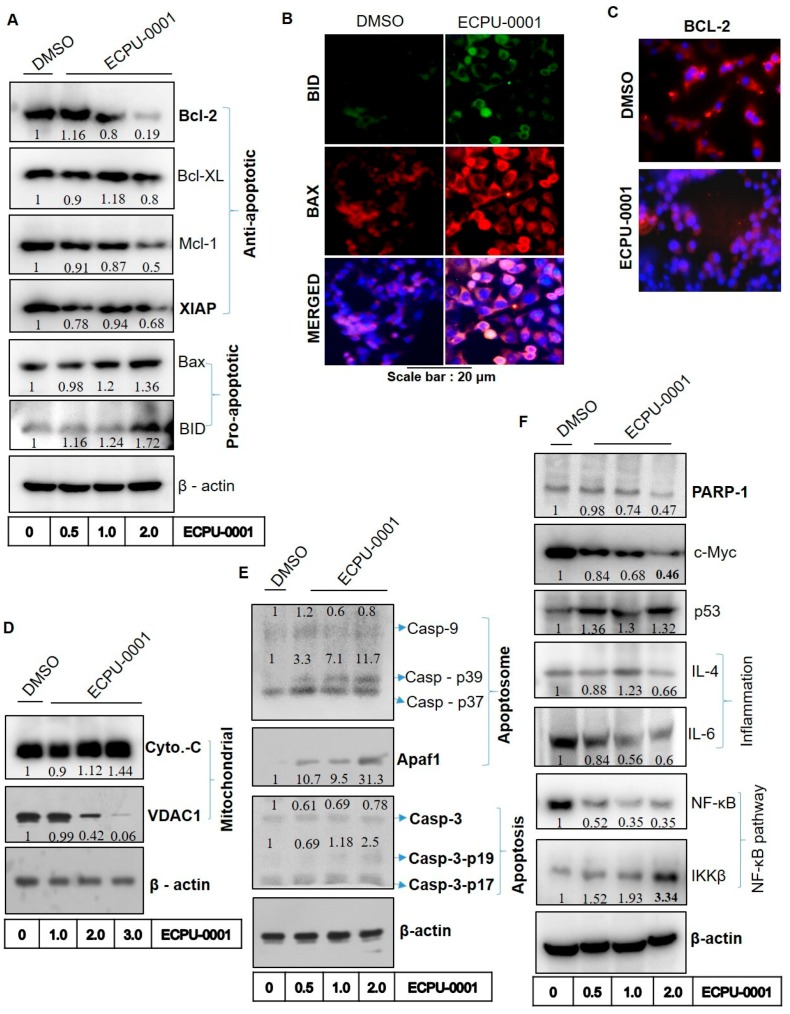
ECPU-0001 inhibits cancer growth of A549 through BCL-2 mediated mitochondrial apoptosis pathway. (**A**) Immunoblot study was done to investigate the protein levels of anti-apoptosis markers as Bcl-XL, BCL-2, XIAP, Mcl-1, apoptosis inducing markers as BID and BAX. (**B**) Immunocytochemistry study performed to evaluate role of ECPU-0001 in activation of BID and BAX. (**C**) Immunocytochemistry study showed significant reduction in BCL-2 protein levels in ECPU-0001 treated A549 cells. (**D**) Protein levels analysis by western blot for mitochondrial proteins Cyto-C and VDAC1. (**E**) ECPU-0001 augments apoptosome formation and showing the activation of caspases proteins. Immunoblot study for apoptosome inducer Caspase-9 and Apaf1 as well as apoptotic protein Caspase -3. Cells were treated with different concentration of ECPU-0001 for 24 h and then harvested. Total cell fractions were collected using RIPA buffer subjected to SDS PAGE followed as previously as described in Materials and Methods. (**F**) Western blot analysis was conducted for DNA repair protein PARP-1, oncogenic protein c-MYC, tumor suppressor protein p53, tumor inflammatory protein IL-4 and IL-6 as well as NF-κB pathway proteins NF-κB and IKKβ.

ECPU-0001 strongly inhibited BCL-2 protein in A549 cells, which was confirmed by our various in-silico as well as in vitro assays such as molecular docking study, MD simulation study, and thermal shift assay. Therefore, we aimed to investigate effect of BCL-2 inhibition on BCL-2 associated intrinsic pathway of apoptosis and in due course, numerous anti-apoptotic as well as pro-apoptotic protein levels were assessed by immunocytochemistry (ICC) and western blot studies. Energy-producing organelles mitochondria play important role in cell life and their programmed cell death in most of tissues under the guidance of BCL-2-family proteins. Our result indicates that ECPU-0001 significantly reduced expression of anti-apoptotic members BCL-2, BCL-2-xL, XIAP as well as Mcl-1 and enhanced levels of pro-apoptotic BID and BAX in western blot analysis (Figure 6A and Figure S4). Over expression of BAX and BID protein was also clearly visualized in ICC analysis (Figure 6B and Figure S4). Significant reduction of BCL-2 protein also observed in ICC study (Figure 6C). Further, we extend our investigation on translocation of Cytochrome-C from mitochondria to cytosol, which is a crucial biomarker of intrinsic pathway mediated apoptosis process. Voltage-dependent anion channel 1 (VDAC1), the most common protein that’s found in the mitochondrial outer membrane. VDAC1 works as a gatekeeper for the several nucleotides, metabolites and ions from mitochondria. As a result, the mitochondrial membrane potential is dissipated that confirmed by immunoblot of mitochondrial membrane protein VDAC1 resulted release of Cytochrome-C (Figure 6D and Figure S5). Various concentrations of ECPU-0001 treatment noticeably enhanced Cytochrome-C at the translational level as compared to control (Figure 6D). Released Cytochrome-C, apoptotic activator factor-1 (APAF-1) and caspase-9 form a complex, known as apoptosome, which induces caspase-3 and pave the way for intrinsic apoptotic pathway [13]. Therefore, we directed to expound effect of ECPU-0001 treatment on APAF-1, caspase-9 and caspase-3 level. Results have illustrated that the ECPU-0001 treatment effectively elevated protein level of APAF-1 compared to control (Figure 6E). ECPU-0001 treatment also effectively raised expression of caspase-9 and caspase-3 protein in immunoblot analysis (Figure 6E, Figure S6). These evidences further evoked participation of ECPU-0001 to induce intrinsic apoptotic pathway by targeting BCL-2. Furthermore, ECPU-0001 has down regulated the caspase activity inhibitor protein XIAP concentration dependently (Figure 6A).

### 2.8. ECPU-0001 Inhibits A549 Cells Growth via Regulation of p53, PARP-1 and c-MYC

PARP-1 is a nuclear enzyme that regulates DNA repair resulted ability to proliferate tumor cells even in the presence of DNA break via upregulation of DNA repair mechanism through PARP-1 [39]. Treatment of ECPU-0001 significantly suppressed expression of DNA repair protein PARP as compared to untreated cells, which may also validate the role of ECPU-0001 on the DNA break induced apoptosis and cell death (Figure 6F and Figure S5). Consequently, we have also examined the effect of ECPU-0001 treatment on apoptotic machinery activator p53, which mediated apoptosis principally by activating intrinsic pathways. Various reports have also shown that p53 activation is accompanied by c-MYC and both have been proven as a decisive factor for cell life [40]. Our results showed that ECPU-0001 treatment significantly raised expression of p53 protein and markedly down regulated c-MYC protein expression in immunoblot analysis (Figure 6F and Figure S7). Accordingly, up-regulation of p53 and down regulation of c-MYC by ECPU-0001 treatment ignited whole apoptotic machinery, which profoundly provoked intrinsic pathway governing apoptosis. These significant results evidently point out that ECPU-0001 induces apoptosis in A549 cells by triggering of the intrinsic pathway of apoptosis.

### 2.9. Effects of ECPU-0001 in Regulation of Oncogenic NF-κB, IL-4 and IL-6

BCL-2 inhibits apoptotic process by regulating genes that translate proteins such as NF-κB, IL-6 and IL-4 [14,15,16]. The nuclear factor-κB (NF-κB) belongs to a family of transcription factors which impart significant role in inflammatory responses, apoptosis and proliferation as well as oncogenesis. Therefore, inhibition of NF-κB and IL-6 induce apoptosis progression in cancer cells and investigated as an imperative target to control cancer progression. Moreover, IKKβ is the catalytic subunit of IκB kinase, which play vital role in activation of NF-κB complex and degradation of IKKβ activates translocation of NF-κB into nucleus which turn on the expression of several genes, lead to provide a physiological response such as inflammation, cell survival and apoptosis [41]. Our western blot analysis revealed that ECPU-0001 treatment significantly down regulated NF-κB as well as IL-6 concentration dependently and up-regulated expression of IKKβ (Figure 6F, Figure S5). IL-4 is a multifactorial cytokine that contributes an important function in the modulation of immune responses. Few cytokines interact with IL4R (especially IL4Rα) results in the enhancement of tumor growth, multiple-drug resistance against apoptosis and differentiation. Therefore, we also elucidate the inhibitory role of ECPU-0001 against IL-4. Our results showed that ECPU-0001 significantly downregulated the protein levels of IL-4 in A549 cells in a dose dependent manner (Figure 6F). These compelling evidences suggested that the apoptotic potential of ECPU-0001 also governed by down regulation of NF-κB, IL-4 and IL-6.

### 2.10. ECPU-0001 Inhibits Tumor Growth and Reduces Tumor Burden in a Xenograft Nude Mouse Model

**Figure 7:**
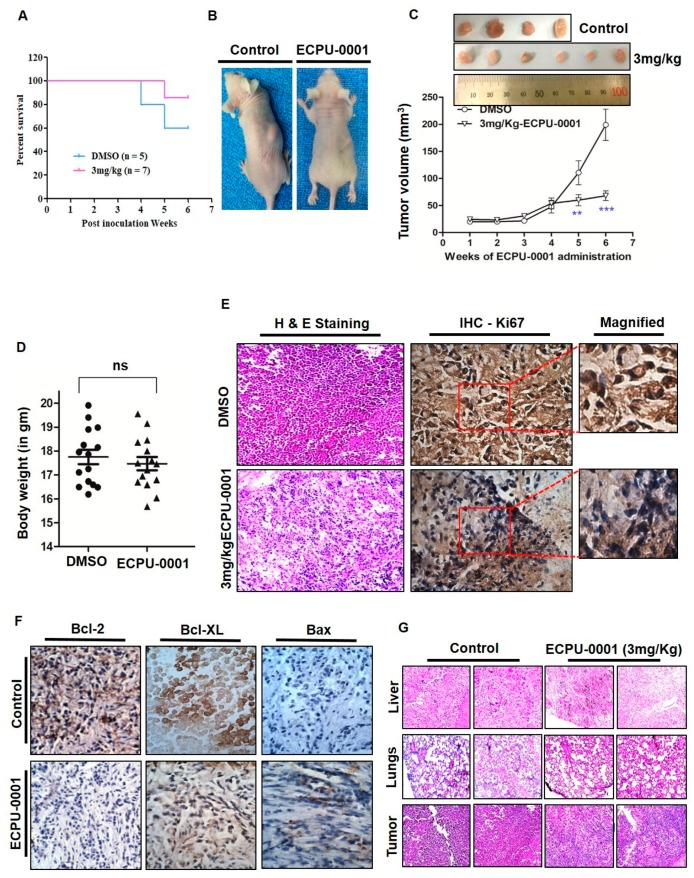
ECPU-0001 inhibits tumor growth and reduces tumor burden in xenograft nude mouse model. (**A**) Mice were euthanized when tumor volume reached 220 mm^3^, and survival is shown in a Kaplan-Meier plot (*p* < 0.05). (**B**) ECPU-0001 inhibits A549-lung cancer tumor growth and metastasis in in vivo subcutaneous xenograft model. (**C**) BALB/c nude mice inoculated with 2.5  ×  10^6^ A549 cells in right/left flanks were administered 3.0 mg/kg of ECPU-0001 or DMSO as a control, two times a weekly for six weeks. Tumor volume was measured every third day. Data shown are mean ± SD (*n* = 7; ** = *p* < 0.01, *** = *p* < 0.001). (**D**) Body (gm) weight was measured once a week during the experiment. (**E**) Immunohistochemistry of oncoprotein Ki67 in tumor sample slides (Magnification: 400X). Tumor-bearing nude mice were administered with ECPU-0001 and DMSO every alternate day for six weeks. Then solid tumors were collected after the mice sacrificed and process for IHC histopathological analysis. Tumor volume and area were recorded from each tumor. Left panel showing ECPU-0001 efficacy in A549 induced tumor sections by H & E staining. (**F**) Tumoricidal activity of ECPU-0001 with minimal cytotoxicity via induction of apoptosis in vivo. Here, elucidated the histopathology of xenograft tumors. The tumor sections were under IHC staining using specific antibody against BCL-2, Bcl-XL and BAX (20× magnification). IHC distinctly showed ECPU-0001 significantly downregulation of BCL-2 and BAX activation in tumor tissues. (**G**) Captured images presenting the tissue sections of lung, liver organs of A549 cell xenograft model on nude mice (top and middle). Scale bar: 400×. On the other hand, H & E staining of tumor tissue in DMSO-treated control group and ECPU-0001-treated group (lower). ECPU-0001 (3 mg/Kg) administration in A549-tumor bearing nude mice showed reduced tumor morphology compared with DMSO treated control group. In addition, there are not only seen metastatic tumor but also ECPU-0001 could not change the morphology of both lung and liver (no damaged liver or lung showed minimal cytotoxicity) compared to control sections.

The in vivo anticancer potential of ECPU-0001 has been evaluated in the lung adenocarcinoma xenograft model with BALB/c-nu nude mouse at the dose 3 mg/kg body weight. After development of successful model, ECPU-0001 has been provided 3 mg/kg for 42 days and control animal were treated with vehicle. Firstly, the survival rate of mice ECPU-0001 treated/untreated was analyzed by the long rank test. The Kaplan-Meier graph demonstrated that ECPU-0001 significantly enhanced survival rate as compared to the untreated group. Treatment with ECPU-0001 was increased more than 20% life span (survival rate = 85.714%) as compared to control (survival rate = 60%) after 42 days treatment (Figure 7A). In addition, ECPU-0001 also reduced tumor volume significantly in time dependent manner as compared to control group (** = *p* < 0.01 at 5th week and *** = *p* < 0.001 at last week) (Figure 7B,C). Further, tumors of mice were removed and weight and it was noticed that ECPU-0001 at 3 mg/kg significantly decreased tumor weight as compared to untreated control. It was also noticed that ECPU-0001 treatment did not reduce body weight of animal as compared to control group which advocate non-toxic behavior of ECPU-0001 (Figure 7D). During the whole experimental duration ECPU-0001 treated animals did not display any type of toxicity related symptoms such as alteration in behavior, food and water intake.

### 2.11. Histopathological Investigation of Xenograft Tumor

Furthermore, for elucidation of ex vivo cell viability and antitumor efficacy of ECPU-0001, an IHC study of Ki67 (proliferation marker for tumor), and H & E staining (for nucleus and cytoplasmic inclusions of tumor tissues) were performed. Figure 7E depicts representative immunohistochemistry for Ki67 and H & E staining of tumor of a control mouse and a tumor of ECPU-0001-treated mouse. The Ki67 expression as well as the mitotic count was significantly lower in the tumors of ECPU-0001-treated mice compared with control. ECPU-0001 treated A549 tumors showed significant reduction in tumorigenic marker Ki67 protein levels in immunohistochemistry study of tumor sections (Figure 7E).

### 2.12. Fabulous Tumoricidal Activity via Induction of Apoptosis In Vivo

Our in vitro study confirmed that ECPU-0001 effectively induce intrinsic pathway of apoptosis by targeting BCL-2. To validate these in vitro result, we also measured expression level of BCL-2 and BAX protein expression in xenograft tumor. We found that BCL-2 level in ECPU-0001 treated group’s tumor was significantly (*p* < 0.05) reduced compared to control group (Figure 7F). One the other hand, expression of pro-apoptotic proteins BAX was significantly increased in the tumor of ECPU-0001 treated nude mice. Thus, immunohistochemistry (IHC) investigation demonstrated that ECPU-0001 treatment clearly reduced expression of BCL-2 and BCL-2-xL, while enhanced expression of BAX (Figure 7F and Figure S7) in tumor.

### 2.13. Toxicity Evaluation

Firstly, toxicity as well as side effects of ECPU-0001 were judged by observing consumption of intake water, behavior, relative body weight and food of the experimental animals on a daily basis. Observation indicated that there was no significant (ns) difference in relative body weight between the ECPU-0001 treated group and the control group (normal mice) were observed (Figure 7G). Additionally, no behavioral fluctuations as well as an alteration in food and water consumption were seen during the whole experimental period. Moreover, H & E staining analysis of liver and lungs showed that there was no significant difference in morphology of liver and lung tissue between the ECPU-0001 treatment group and the control group (Figure 7E,G). Thus, these investigations clearly suggested ECPU-0001 treatment was not overtly toxic for the mice.

## 3. Discussion

The discovery and development of anticancer drugs, especially cytotoxic agents are a challenging task and it differs vastly from the drug development strategy for any other diseases [42]. Current therapy of cancer is suffering from numerous obstacles such as severe side effects, normal cell cytotoxicity as well as anticancer drug resistance [42]. Thus, discovery of safe novel chemical entities which may provide a safer option to control cancer is always appreciable. In this regard, we have designed and synthesized a novel hybrid molecule ECPU-0001 and their extensive anticancer efficacy along with the mechanism of action has been elucidated in vitro as well as in vivo.

BCL-2 proteins are considered as the main regulator of apoptosis [14]. Anti-apoptotic BCL-2, Bcl-XL as well as MCL-1 vanquishes apoptosis by stopping Cytochrome-C migration to cytoplasm [14]. Even, BCL-2 participates in the apoptosis and programmed cell death via upregulation of pro-apoptotic member such as BAX, BAK and BID [14,15]. Previous study showed that BCL-2 inhibitor Venetoclax and navitoclax enhanced apoptosis in vitro and reduced tumor propagation or burden in BCL-2–expressing lung cancer cells induced animals in vivo [16,17,18]. Our in-silico and in-vitro study confirmed that ECPU-0001 strongly interacts with BCL-2 protein to induce apoptosis in A549 cells. ECPU-0001 exhibited moderate to excellent cytotoxic effect against a panel of cell line. In addition, ECPU-0001 showed remarkable cytotoxicity against A549 cells in a different dose of ECPU-0001 as compared to other cell lines with an IC_50_ value 1.77 µM. FACS analysis showed that ECPU-0001 displayed promising apoptotic effect in A549 cells. A significant early and late apoptosis was also observed in FACS analysis after dual staining with Annexin-V-FITC and PI. Further, ECPU-0001 arrested G2/M phase check point of A549 cells by targeting CyclinD1/CDK6, which pinpointing apoptosis induce by ECPU-0001 also involving its cell cycle arrest ability. In cell migration assay ECPU-0001 significantly inhibit migration of A549 cells concentration dependently.

To investigate effects of BCL-2 inhibition on other biomarkers of the intrinsic apoptotic pathway, ICC and immunoblot study were performed. Interestingly, treatment with ECPU-0001 significantly down regulated BCL-2 at translational levels. ECPU-0001 also modulates Bcl-XL as well as MCL-1 protein and up regulated BAX, BAK and BID protein at translational level leading to increased Cytochrome-C Level. Immunoblot analysis visibly indicates an increased level of Cytochrome-C as compared to untreated cells. This cascade changing produced by ECPU-0001 is indicative of its mechanism to produce apoptosis through the intrinsic pathway by targeting BCL-2. Further, in the intrinsic pathway of apoptosis, released mitochondrial Cytochrome-C binds with APAF-1 and caspase-9 (to form apoptosome) which further activates caspase-3 and lead to precede apoptosis [43,44]. Similarly, ECPU-0001 treatment significantly increased APAF-1, caspase-9 and caspase-3 levels, which further confirm its involvement in the intrinsic pathway of apoptosis. Apart from that ECPU-0001 also significantly suppressed expression of PARP-1 which is regarded as a prime regulator of the execution phase of DNA repair and associated with an intrinsic pathway [39]. The X-linked inhibitor of apoptosis protein (XIAP) is directly linked with the intrinsic apoptosis pathway [45]. XIAP prevent apoptotic cell death by inhibiting caspases-3, caspase-7 as well as caspase-9 activities and regarded as a key regulator of the intrinsic apoptosis pathway [46]. Our immunoblot analysis also confirms that ECPU-0001 significantly down regulate expression of XIAP concentration dependently which furthermore suggestive involvement of ECPU-0001 in the intrinsic pathway of apoptosis.

Side by side, we aimed to determine the expression of p53 upon ECPU-0001 treatment because they function as a controller of the apoptotic process by modulating key control points of intrinsic pathway [47]. It is also well studied that p53 regulates most of the protein, which are involved in apoptosis such as BCL-2, BAX, APAF-1, Cytochrome-C migration and caspases [40,47,48]. Further, p53 loss in breast carcinoma induces activation of c-MYC [40]. ECPU-0001 treatment visibly increased p53 levels which have been confirmed by western blot analysis. Additionally, it was also observed that ECPU-0001 significantly down regulates c-MYC expression in immunoblot analysis. Such over expression of p53 along with down regulation of c-MYC after ECPU-0001 treatment further prove the activation of the entire series of events which are associated with apoptosis through the mitochondrial pathway. NF-κB is a well-known nuclear factor stimulated by various carcinogens and tumor promoters and it is a crucial player in tumor progression [49]. NF-κB activation encourages cell proliferation and its suppression led to stop proliferation. NF-κB also maintains the regulation of the BCL-2 family proteins and sustains the mitochondrial integrity that is desirable for the survival of cancer cells [50]. Hence, suppression of NF-κB activity is helpful for induction of apoptosis and studied as an important target to produce apoptosis in cancer cells [50]. The translational level of studies was witness of NF-κB suppression after ECPU-0001 treatment. Additionally, ECPU-0001 also down regulated cytokines IL-6 and IL-4 level, which plays an important link between inflammation initiation and progression of oncogenesis [51]. Moreover, ECPU-0001 also up-regulated IKKβ, a main regulatory protein for activation of NF-κB. These studies provided significant evidence that ECPU-0001 also participates in NF-κB-IL-6 signaling pathway to kill A549 cells. ECPU-0001 exhibited in vivo antitumor efficacy of in A549-xenograft nude mice model and our result demonstrated that ECPU-0001 significantly enhanced survival time in tumor bearing animals as compared to untreated animals. ECPU-0001 at the dose of 3 mg/kg effectively reduced tumor weight and tumor volume as compared to untreated animals. In an IHC study of xenograft tumors, ECPU-0001 significantly suppressed expression of BCL-2 and BCL-2-xL, while enhanced expression of BAX as similarly found in vitro study in A549 cells. Additionally, IHC investigation also revealed that ECPU-0001 treatment reduced Ki67 expression as well as mitotic count as compared to the untreated group of animals. Thus, studies signify that ECPU-0001 have the immense capability to slow down tumor progression in A549 xenograft nude mice, which led to the enhanced life span of animals. These in vivo antitumor efficacies of ECPU-0001 further supports its in vitro anticancer activity against A549 cells.

## 4. Materials and Methods

### 4.1. Docking Study

We performed molecular docking to illustrate the protein-ligand interaction of ECPU-0001 using AutoDock 4.2 [52]. The atomic coordinates of BCL-2 (PDB ID: 6GL8) was taken from Protein Data Bank (www.rcsb.org). All the hetero atoms were removed, a missing region in pdb file was connected as a loop using Chimera and the structure of protein was optimized. Both, the protein and ligand files were prepared and saved in PDBQT format which were used as initial input for AutoDock run following the standard protocol. The Lamarckian genetic algorithm (LGA) method was applied to carry out docking [53].

### 4.2. Molecular Dynamics (MD) Simulation Analysis

All-atoms MD simulation was performed on the atomic coordinates BCL-2 and BCL-2-ligand complex using the biosimulation package GROMACS v5.1.4 with CHARMM27 as force field [54]. Two independent runs were set for BCL-2 and BCL-2-ligand complex. The system was solvated with TIP3P water model in dimension of cubic box (6 nm^3^) and 0.15 M of counter ions (Na^+^ and Cl^–^ ions) were added in solvent to neutralize the overall charges. The energy minimization of prepared system was done by the 50,000 iterations of steepest descent. And, we applied periodic boundary condition in x-, y- and z-dimensions. The equilibration of system involved run of 500 ps for each NVT and NPT ensemble, respectively. During the simulation, temperature and pressure were taken care by Berendsen thermostat [55] and the Parrinello-Rahman pressure [56], respectively. LINC algorithm was applied to constrain the atomic bonds and angles [57]. The long-range electrostatic interactions were defined with particle mesh Ewald (PME) [58] and LJ potential (with cutoff 0.10 nm) applied for van der Waals interactions. Using, NPT ensemble the production runs for period of 200 ns was performed on GPU enabled Intel i7-x86-64 machine with OS Centos 7 [59]. The integration time step during the simulation was fixed to 2 fs and trajectories were recorded for each 5 ps. The obtained MD trajectories were analyzed with GROMACS utilities available with software.

### 4.3. Chemistry: Synthesis and Characterization of ECPU-0001

All the starting material and dried organic solvents were acquired from TCI Chemicals (Tokyo, Japan), Sigma Aldrich (St. Louis, MO, USA.) and Merck (Darmstadt, Germany). ^1^H and ^13^C nuclear magnetic resonance (NMR) spectra were recorded with a high resolution Jeol NMR spectrophotometer (Jeol Inc., Peabody, MA, USA) at 400 and 100 MHz, respectively. Chemical shifts are presented in parts per million (ppm) comparative to the solvent peak or tetramethylsilane (TMS). The coupling constants (J) are described in hertz (Hz), and the splitting patterns are indicated by using abbreviations: s (singlet), d (doublet), t (triplet), m (multiplet), brs (broad singlet), and dd (double doublet). LC/MS data were obtained by an Agilent 6310 Ion trap LC/MS system (Santa Clara, CA, USA). Melting point were taken in open capillaries by using the model KSPII, from KRUSS (GmbH, Hamburg, Germany). Purity of the ECPU-0001 was analyzed on a Shimadzu HPLC system (Kyoto, Japan) united with a photodiode array detector (PDA) and C-18 column. ECPU-0001 have shown 99% HPLC purity. 

#### 4.3.1. Procedure for the Synthesis of 1-(2-Chloro-ethyl)-3-(9-ethyl-9H-carbazol-3-yl)-urea(2)

An equimolar amount of carbazole 1 and 2-chloroethyl isocyanate were stirred in THF for 12h at 0–5 °C. Upon completion of reaction, the reaction mixture was diluted with water and appeared precipitate was filtered and washed twice with water and petroleum ether to yield pure intermediate 2 in good yield.

#### 4.3.2. 1-(2-Chloro-ethyl)-3-(9-ethyl-9H-carbazol-3-yl)-urea (2)

White solid; yield 74%; mp 171–173 °C. 1H-NMR (400 MHz, DMSO-D6) δ 8.58 (s, 1H), 8.17 (s, 1H), 8.00 (d, J = 7.8 Hz, 1H), 7.34–7.51 (m, 4H), 7.11 (t, J = 7.3 Hz, 1H), 6.37 (t, J = 5.5 Hz, 1H), 4.34 (q, J = 6.9 Hz, 2H), 3.65 (t, J = 6.2 Hz, 2H), 3.43 (t, J = 5.7 Hz, 2H), 1.24 (t, J = 6.9 Hz, 3H); LC−MS: m/z; 316 (M + 1).

#### 4.3.3. Procedure for the Synthesis of 1-(9-Ethyl-9H-carbazol-3-yl)-3-{2-[4-(furan-2-carbonyl)-piperazin-1-yl]-ethyl}-urea (ECPU-0001)

Chloroethyl urea derivative 2 and 2-furoyl piperazine (equimolar ratio) were dissolved in THF, catalytic amount of NaI and NaHCO_3_ were added into the reaction mixture and refluxed for 14 h. The reaction mixture was diluted with water and product was extracted with ethyl acetate. The organic layer was evaporated under reduced pressure and obtained crude product was purified by column chromatography using chloroform and methanol as an element (96:4) to achieve target compound ECPU-0001.

#### 4.3.4. 1-(9-Ethyl-9H-carbazol-3-yl)-3-{2-[4-(furan-2-carbonyl)-piperazin-1-yl]-ethyl}-urea (2)

White solid; yield 84%; mp 154–156 °C. δ 1H-NMR (400 MHz, DMSO-D6) δ 8.56 (s, 1H), 8.20 (s, 1H), 8.03 (d, J = 7.8 Hz, 1H), 7.83 (s, 1H), 7.36–7.53 (m, 4H), 7.13 (t, J = 7.3 Hz, 1H), 6.98 (d, J = 3.2 Hz, 1H), 6.61 (t, J = 1.6 Hz, 1H), 6.06 (t, J = 5.0 Hz, 1H), 4.37 (q, J = 6.9 Hz, 2H), 3.63 (d, J = 34.8 Hz, 4H), 3.26 (q, J = 6.0 Hz, 2H), 2.43–2.49 (m, 6H), 1.27 (t, J = 7.1 Hz, 3H); 13C-NMR (100 MHz, DMSO-D6) δ 158.7, 156.2, 147.5, 145.2, 140.4, 135.7, 133.1, 126.0, 122.6, 120.6, 118.8, 118.7, 116.0, 111.8, 110.4, 109.4, 57.9, 53.3, 40.6, 37.4, 36.8, 14.2, LC−MS: m/z; 460 (M + 1). HPLC purity: 99%.

### 4.4. Cell Lines and Culture Conditions 

Cell lines A549, NCI-H1299, MCF-7, HT-29, U2OS and Hela were procured from ATCC (Manassas, VA, USA). NCI-H1299 cells were grown in RPMI-1640 complete medium (WelGENE, Inc., Gyeongsangbuk-do, South Korea). The preparation of complete RPMI-1640 medium is followed with the addition of 10% fetal bovine serum (FBS; Hyclone, Logan, UT, USA) and 1% Antimycotic & Antibiotic (cat. No.15240-06; Gibco, Gaithersburg, USA). While MCF-7, A549, U2OS, Hela and HEK293T cells were grown in DMEM complete medium. The DMEM complete medium was supplemented with 10% FBS (Hyclone), 2 mmol/liter glutamine, and 1% penicillin and streptomycin. The cells were propagated under a steady state conditioned 5% CO_2_, 95% air and at the 37 °C. 

### 4.5. Cellular Thermal Shift Assay (CETSA) and BCL-2-Targeting Assay

The ability of ECPU-0001 to interact with in-cell BCL-2, thereby stabilize the target in intact cells, was performed and analyzed essentially as described [38,60]. Summarily, A549 cells were cultured and exposed with ECPU-0001 for 2 h. Then cells were harvested by the moderate speed of centrifugation and washed twice with cold PBS. After that, cells were diluted in 1× cold PBS. To extract protein resuspended cells were thawed-freeze four times in −80 °C. The protein lysate was separated and collected using spin-down at 12,000 rpm for 20 min at 4 °C. The BCL-2-ECPU-0001 interaction and thermal shift were analyzed by immunoblots analysis.

### 4.6. Cytotoxicity and Proliferation Assay

The cell cytotoxicity was investigated by Trypan-Blue exclusion method. Firstly, cells were harvested and stained with Trypan-blue (EBT-001-NanoEnTek-Inc., Gyeonggi-do, South Korea) followed the same conditions for all samples. The ratio of cells versus dye was 10:10 i.e., 10 μL cells (1 × 10^5^ cells) and 10 μL Trypan-Blue then mixed in 1.5 mL Eppendorf tubes in a clean bench. The mixed cells were loaded onto EVE^TM^ slides (Nano-EnTek-Inc.). To investigate the number of total cells, live-cells and dead-cells followed a previous study [61]. Cell cytotoxicity percentage was calculated by using percentage formula for live-cells in the treated group versus live cells in control group in different concentration of ECPU-0001 (0, 1, 2, 3 and 4 μM ECPU-0001 and DMSO) after culturing in complete media (CM). Efficacy of ECPU-0001 and morphological analysis were also observed by optical microscope and captured cells morphology images in a different dose.

### 4.7. Early and Late Apoptosis Analysis

A549 cells harvested followed by trypsinization (0.25% EDTA-Trypsin, Thermo Fisher Scientific Korea Co., Ltd., Gangnam-gu, South Korea), the cells were diluted in 3 mL RPMI complete medium and cultured in 60 mm cell culture dishes for overnight. Next day, again cells were 1 × 10^6^ cells harvested and applied rinsing twice with 1× cold PBS. To investigate pre-apoptosis and late apoptosis, cells were stained with 5 μL of Annexin V and propidium Iodide (PI) gently swirl to proper-mix and then incubated in room temperature for 30 min in the dark followed Annexin V/FITC apoptosis detection kit (AntGene, ant003, Wuhan, China) guidelines. Added 500 μL 1× binding buffer and mixed gently. Cells were analyzed for apoptosis using FACS caliber (Becton-Dickinson, San Jose, CA, USA).

### 4.8. Cell Cycle Checkpoints Analysis by FACS

The distribution of cells in different checkpoints was analyzed by staining with propidium Iodide (PI). And the analysis was carried out by the BD FACSCaliburTM system followed procedure as mentioned previously [41]. Following to the treatment with 0.5 μM, 1.0 μM and 2.0 μM concentrations of ECPU-0001 for 24 h, the A549 cells were collected approximately ~1 × 10^6^ cells/mL. The fixation of cells with 70% ethanol was performed at room temperature by gradual vortex (slowly and gently) and incubated at 4 °C for 24 h. The fixed A549-cells were washed twice and followed the protocol as described in a previous study [41]. The propidium iodide- stained cell signals were observed by the FL2-channel and the ratio of DNA level in the different cell cycle stages was calculated using the Modfit LT Software (Topsham, ME, USA).

### 4.9. Immunocytochemistry 

Immunocytochemistry and immunofluorescence study of BCL-2, BID and BAX were performed as reported previously [41]. A549 cells were fixed and stained with BCL-2, BAX and BID antibodies. Immunofluorescence and expressed proteins were imaged as well as analyzed with an inverted fluorescent microscope Zeiss Axiovert 200 M (Carl Zeiss, Thornwood, NY, USA).

### 4.10. Protein Extraction and Immunoblot Analysis

A549 cells (approximately ~5 × 10^6^ cells/mL) were grown in complete medium with 1 to 3 μM concentration of ECPU-0001 and control cells were treated with DMSO. Next day, cells were collected by centrifuge (Hanil Scientific, Inc., Gyeonggi-do, South Korea) at 1200 rpm for 3 min at 4 °C. Cells were rinsed with 1× cold PBS. The pellet was downed and harvested cells were lysed in cell lysis RIPA buffer containing DTT, NaVO3 and PI respectively. After quantification of protein concentration using the Bradford method, the lysate was mixed with 4× protein sample buffer then boiled in 95 °C boiling water bath for 10 min followed entire steps as mentioned elsewhere [61]. The membranes were immunoblotted with the following antibodies: mouse monoclonal anti-BAX, anti-VDAC1, anti-c-MYC, anti-BID, anti-BCL-2, anti-Mcl-1, anti-Bcl-XL, anti-APAF-1, anti-NF-κB, anti-Iκκβ, anti-caspase-3, and anti-Caspase-9, rabbit polyclonal anti-CytoC, anti-XIAP, anti-IL-4, anti-PARP-1 and anti-BAK. The information of the supplier and dilution for each antibody have provided in the table (Table S1). The PVDF membranes were put at 4 °C in rotating shaker for counter immunoblotted with the anti-mouse and anti-rabbit IgG (1:2500 dilution, Santa Cruz Biotechnology Inc., Delaware Ave, Santa Cruz, CA, USA). Each protein bands were detected by chemiluminescence enhancing solution.

### 4.11. Xenograft Development and ECPU-0001 Efficacy in Tumor-Bearing Nude Mice

5 weeks old BALB/c-nu immunodeficient nude mice were procured from ORIENT BIO Inc. Gyeonggi-do, Korea. The mice were given favorable nutritional conditions and suitable environmental. At the end of experiment, mice were sacrificed at 42th day after ECPU-0001 administration, according to the standard protocols of the Sookmyung Women’s University. The designed research proposal and procedures were sanctioned by the board of reviewing committee (SMWU-IACUC-1701-043-02) of the Department of Animal Facility, Sookmyung Women’s University, Chungparo 47, Yongsan-gu, Seoul 14310, Korea. To investigate the antitumor efficacy of ECPU-0001, a xenograft model was developed as described previously [50]. In order to design the tumor animal experiment, 15 nude mice were divided into three sub-groups. A first group used as test group ECPU-0001 dose (*n* = 7, 3 mg/kg). The second group was A549 cells inoculated non-treated tumor group as a negative control (*n* = 5). The third group was considered as a control group (normal mice, *n* = 3). To induce a tumor, 2.5  ×  10^6^ cells A549 cells were injected subcutaneously into lower right flanks of mice by needle syringe (27 G/12.7 mm, Ultra-Thin Plus™ Korea, Seoul, South Korea). After the generation of successful tumor model ECPU-0001 (3 mg/kg) treatment were administered up to 42 days. When tumors were palpable and visible, the tumors volume were calculated as Volume = ½ × length × Width^2^.

### 4.12. Migration and Wounding Healing Assay

3 × 10^4^ A549 cells were cultured in gelatin (0.1%) pre-coated 6-well flat-bottom plates. To make wounds and scratches, the entire protocol followed as described elsewhere [62]. Briefly wounding, 200 μL sterilized tip was fixed with the pipette and scratched under the same condition to each well. The same protocol has been opted for all wells. After scratched, wells were washed with sterilized PBS and medium respectively. After that, fresh serum-free medium was added to each well for control or with medium containing 2 μM ECPU-0001 to cells and then exposed for 24 h. The area of closing wounds was determined by software ImageJ (http://rsb.info.nih.gov). In contrast, polygon-selection was used to determine the area of closing wound.

### 4.13. Immunohistochemical (IHC) Study

The histopathological analysis of ECPU-0001 was done by IHC. Briefly, sacrificed tumor samples were fixed and embedded in a wax block. From each paraffin-embedded sample, sections were cut in 5 μm by a microtome. Then deparaffinized and rehydrated were done in different percentage of xylene and alcohol respectively. Slides were treated and antigen was retrieved with 3% of hydrogen peroxide in methanol. Slides were blocked and exposed with rabbit polyclonal antibodies for either BCL-2 (diluted, 1:300; Mouse, Cell Signaling, Danvers, MA, USA), Bcl-XL (diluted, 1:500; Mouse, Santa Cruz) or Ki67 (diluted, 1:400; Goat poly, Santa Cruz) or BAX (diluted, 1:500; Mouse, Santa Cruz). Protein expression was observed by biotinylated goat anti-rabbit, anti-mouse IgG (Vector Laboratories, Burlingame, CA, USA). The diaminobenzidine (Vector Laboratories) was used as a substrate, which shows an insoluble brown precipitate. Nuclei were counterstained with Mayer’s Hematoxylin blue. Localized proteins images were captured and processed by the Multistain Imaging Module for BCL-2, BAX, Ki67 and Bcl-XL.

### 4.14. Statistical Analysis 

The data-samples were analyzed for statistically significance using the GraphPad Prism5 software (GraphPad Software, San Diego, CA, USA). Data are collected and used as mean ± SD. The one-way analysis of variance (ANOVA) test was used to determine statistical differences and *p* < 0.05 was considered as a statistically significant.

## 5. Conclusions 

**Figure 8:**
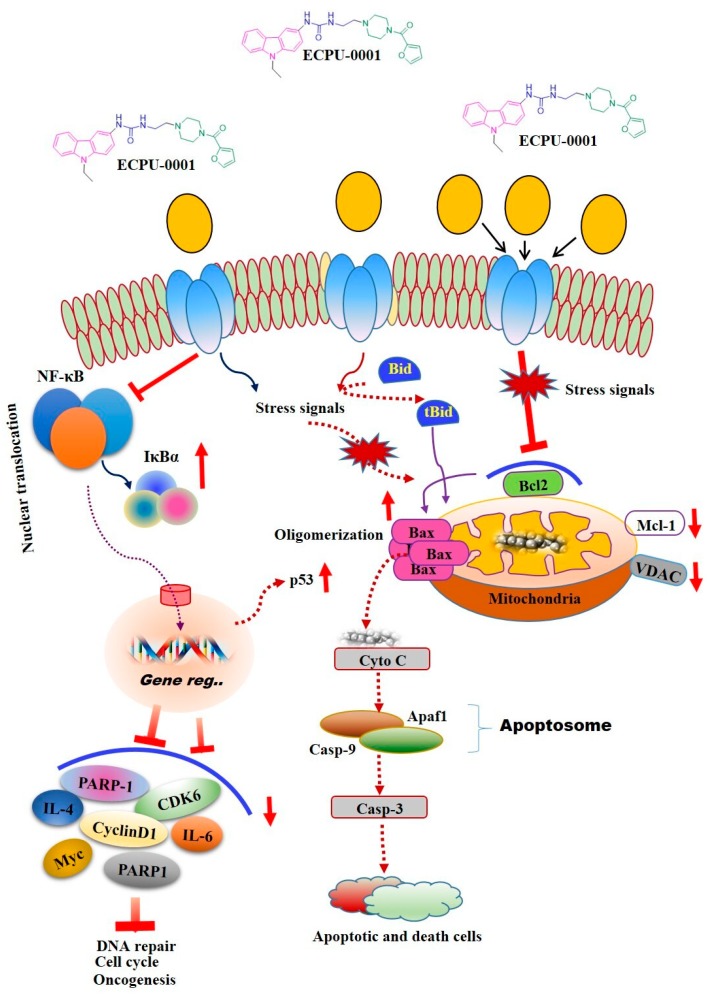
Proposed model for effects of ECPU-0001 in regulation of mitochondrial mediated apoptosis, NF-κB pathway as well as tumorigenic immunomodulators IL-4 and IL-6. Schematic representation of ECPU-0001 efficacy and induction of mitochondrial mediated apoptosis pathway.

Novel small molecule ECPU-0001 appeared as a potent BCL-2 targeting agent which displayed very effective cytotoxicity against A549 cells. Molecular mechanism investigation revealed that ECPU-0001 efficiently targeted BCL-2 to induced apoptosis via intrinsic pathway in A549 cells where it upregulates/down-modulates a number of proteins linked with intrinsic pathway (Figure 8). Additionally, it significantly impedes cancer growth and migration via CyclinD1/CDK6 mediated cell cycle G2/M checkpoint arrest. This molecule also significantly inhibits colony formation of A549 cells and exhibited excellent in vivo anticancer activity against the xenograft mouse model. Moreover, ECPU-0001 effectively control tumor progression in the lung adenocarcinoma xenograft model with BALB/c-nu nude mouse at the dose 3 mg/kg body weight, without exerting severe toxicity. Hence, this investigation provided a novel and potent BCL-2 targeting agent which possesses promising anticancer activity against lung adenocarcinoma with admirable in vivo efficacy.

## Supplementary Materials

The following are available online at https://www.mdpi.com/2072-6694/11/9/1245/s1, Figure S1: RMSF plot of Bcl2 (Black) and Bcl2-ligand complex (Red), Figure S2: Secondary structure of Bcl2, Figure S3: Secondary structure of Bcl2-ligand, Figure S4: Western blot images with molecular weight for CDK6, CyclinD1, BCL-2, Bcl-XL, Mcl-1, XIAP, Bax, Bid and respective β-actin control bands shown in Figure 4G and Figure 5A Figure S5: Western blot images with molecular weight for Cytochrome-C, VDAC1, Caspase-9, Caspase-3, APAF1 and the respective β-actin control bands shown in Figure 5D,E, Figure S6: Western blot images with molecular weight for PARP1, c-Myc, p53, IL-4, IL-6, NF-kB, IKKβ and the respective β-actin control bands shown in Figure 5F. The red dotted rectangle indicates the area shown in Figure 5F, Figure S7: Inhibition of Bcl-2 protein in vivo and immunohistomistry study Table S1: List of antibodies.

## Abbreviations

NSCLC	Non-small cell lung cancer
APAF1	Apoptotic protease activating factor 1
XIAP	X-linked inhibitor of apoptosis protein
CDK6	Cyclin-dependent kinase 6
NF-kB	nuclear factor kappa-light-chain-enhancer of activated B cells
DMSO	Dimethyl sulfoxide
CETSA	Cellular thermal shift assay
VDAC1	Voltage-dependent anion-selective channel 1
IKKβ	inhibitor of nuclear factor kappa-B kinase subunit beta
