# Supplementary material for: Novel Carbazole-Piperazine Hybrid Small Molecule Induces Apoptosis by Targeting BCL-2 and Inhibits Tumor Progression in Lung Adenocarcinoma In Vitro and Xenograft Mice Model
Source: Cancers (Basel). 2019 Aug 25;11(9):1245. doi: 10.3390/cancers11091245 (PMC6770606; doi:10.3390/cancers11091245)
Supplement: Supplementary file 1 [file cancers-11-01245-s001.docx]

Supplementary Materials: Novel Carbazole-piperazine Hybrid Small Molecule Induces Apoptosis by Targeting BCL-2 and Inhibits Tumor Progression in Lung Adenocarcinoma *In vitro* and Xenograft Mice Model

Raj Kumar Mongre, Chandra Bhushan Mishra, Amresh Prakash, Samil Jung, Beom Suk Lee,Shikha Kumari, Jin Tae Hong and Myeong-Sok Lee


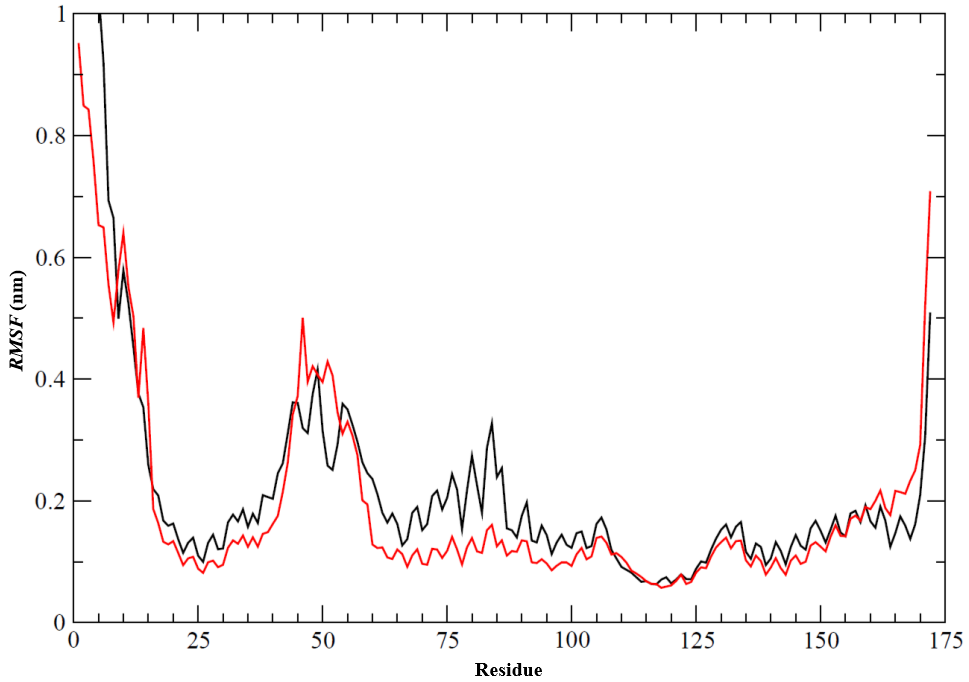


**Figure S1.** RMSF plot of Bcl2 (Black) and Bcl2-ligand complex (Red).


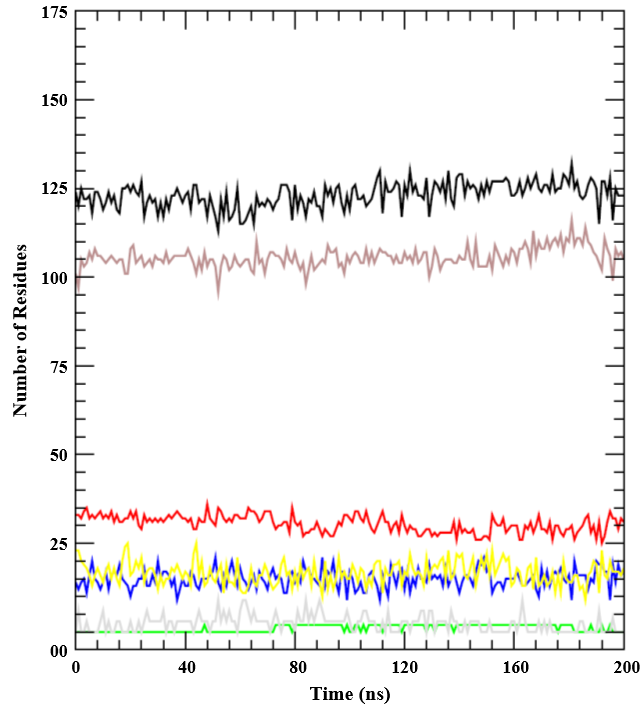


**Figure S2.** Secondary structure of Bcl2.


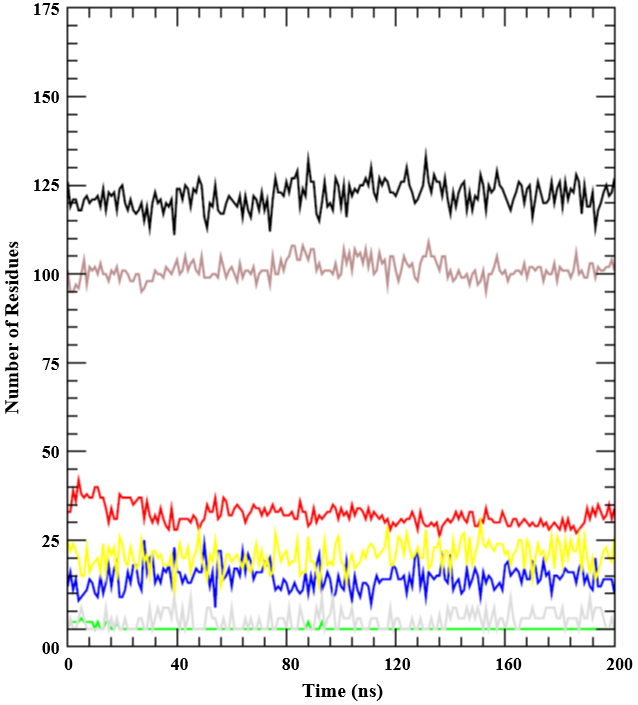


**Figure S3.** Secondary structure of Bcl2-ligand.


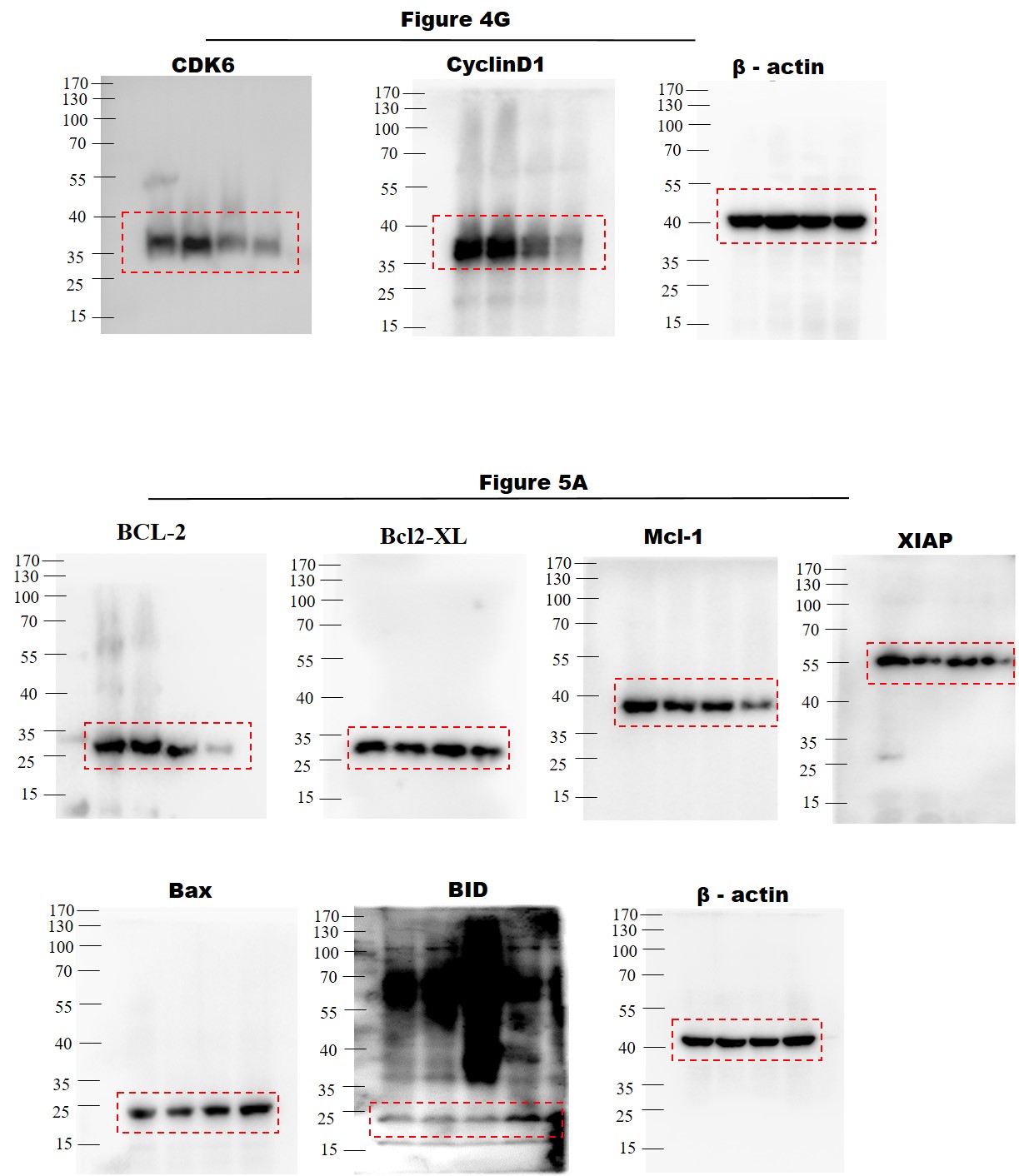


**Figure S4.** Western blot images with molecular weight for CDK6, CyclinD1, BCL-2, Bcl-XL, Mcl-1, XIAP, Bax, Bid and respective β-actin control bands shown in Figure 4G and Figure 5A. The red dotted rectangle indicates the area shown in Figure 4G as well as Figure 5A.


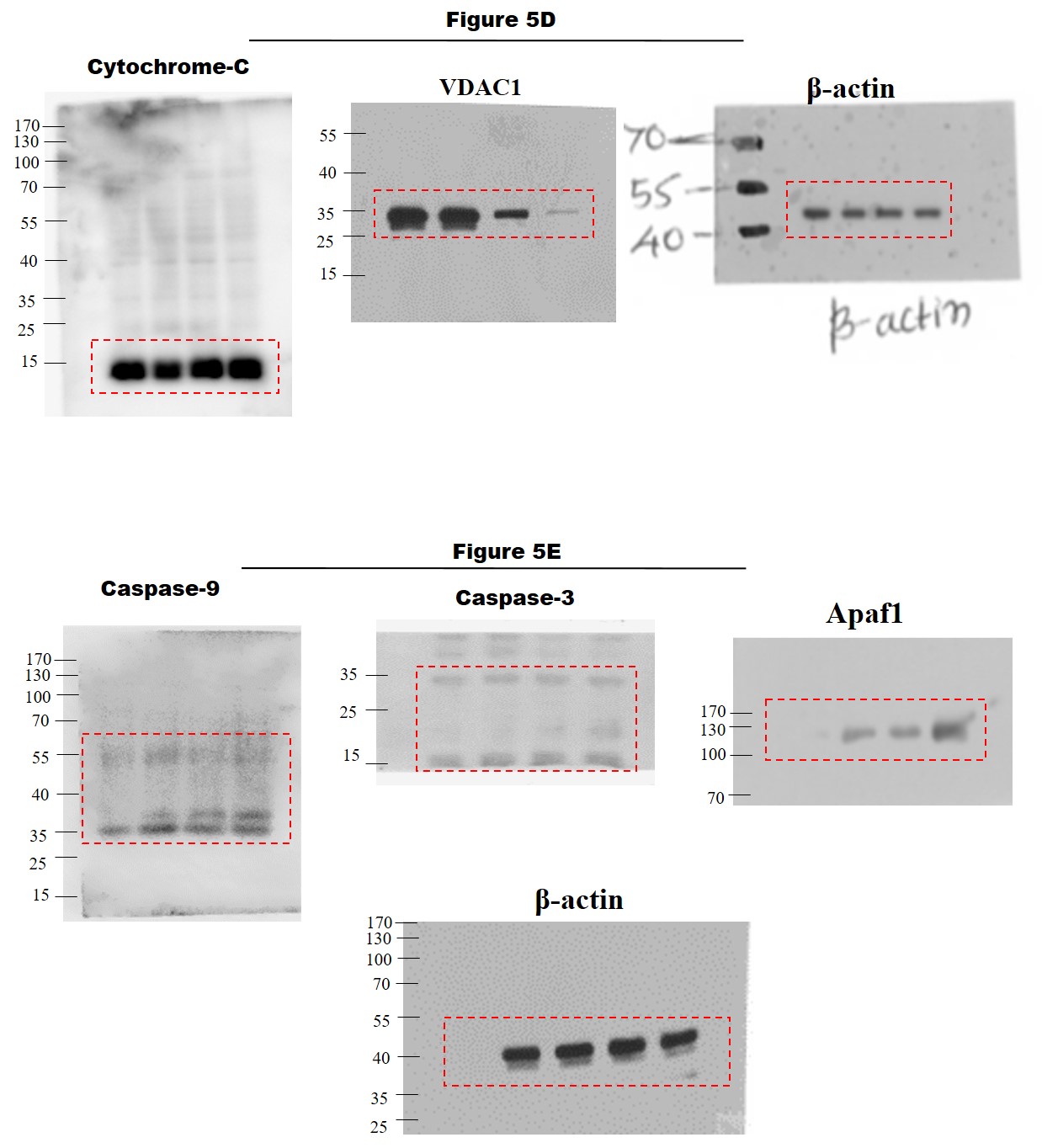


**Figure S5.** Western blot images with molecular weight for Cytochrome-C, VDAC1, Caspase-9, Caspase-3, APAF1 and the respective β-actin control bands shown in Figure 5D and 5E. The red dotted rectangle indicates the area shown in Figure 5D and 5E.


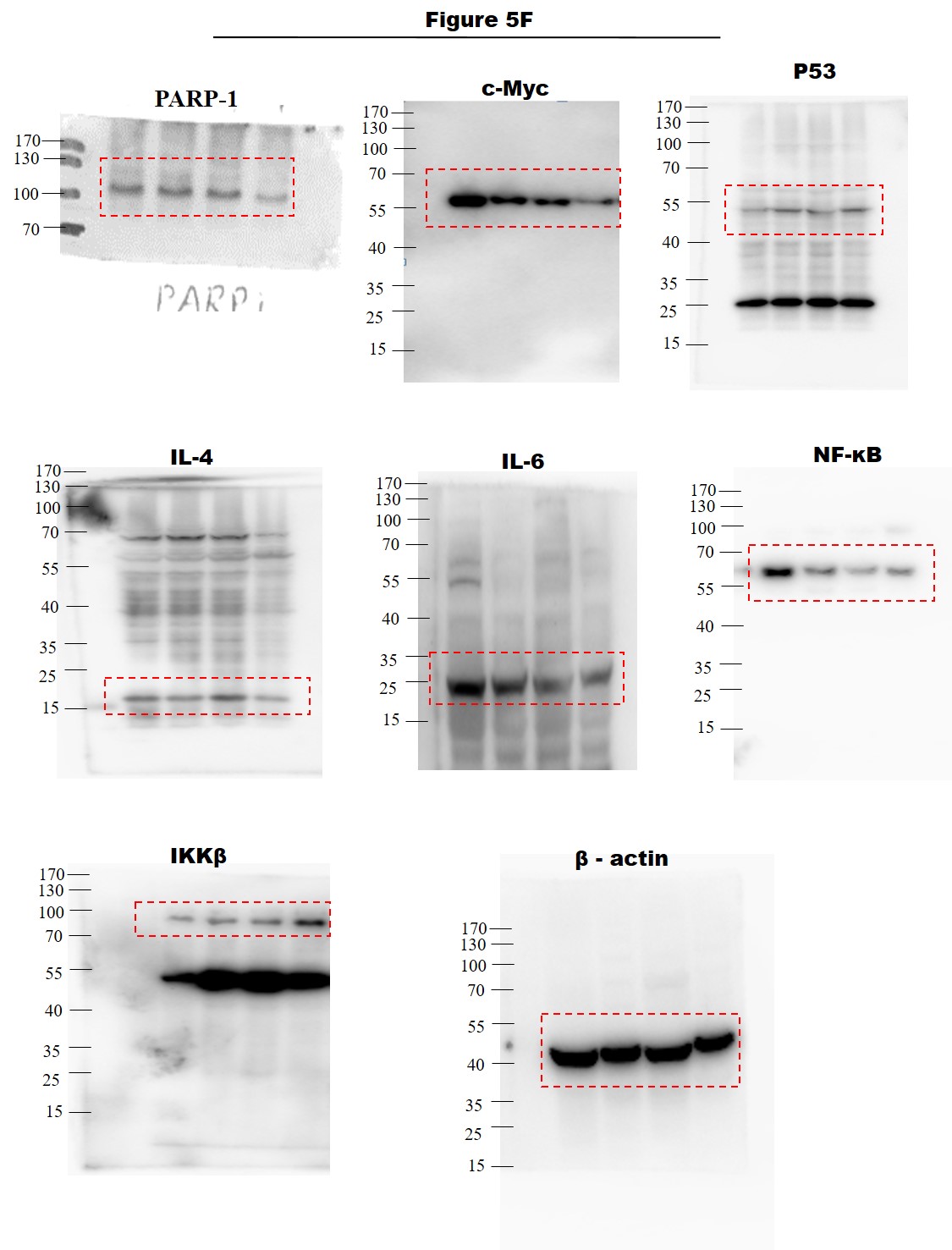


**Figure S6.** Western blot images with molecular weight for PARP1, c-Myc, p53, IL-4, IL-6, NF-kB, IKKβ and the respective β-actin control bands shown in Figure 5F. The red dotted rectangle indicates the area shown in Figure 5F.


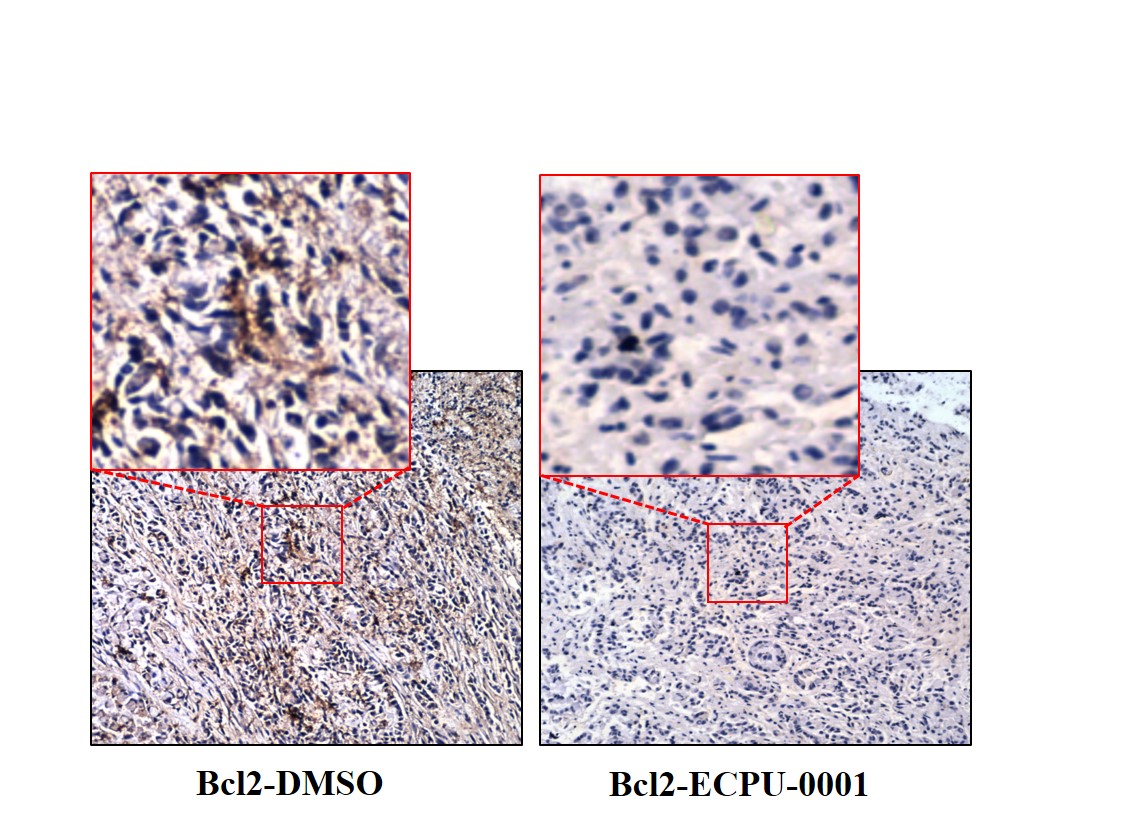


**Figure S7.** Inhibition of Bcl-2 protein in vivo and immunohistomistry study. IHC study showed ECPU-0001 significantly inhibits protein expression of Bcl2 in A549 induced xenograft tumor, similarly, Bcl-2 protein inhibited in A549 cells in-vitro. Scale bar: 400X.

**Table S1.** List of antibodies.

| **Antigens** | **Species Antibodies Raised in** | **Dilution (WB)** | **Supplier** |
| --- | --- | --- | --- |
| Bax | Mouse, monoclonal | 1:500 | Santa Cruz Biotechnology Cat. #sc-7480 |
| Bak | Rabbit, Polyclonal | 1:500 | Santa Cruz Biotechnology, Cat. #SC-832 |
| VDAC1 | Mouse, monoclonal | 1:1000 | Santa Cruz Biotechnology, Cat. #SC-390996 |
| PARP-1 | Rabbit, polyclonal | 1:1000 | Cell Signaling Technology, USA #9542 |
| CDK6 | Mouse, monoclonal | 1:500 | Santa Cruz Biotechnology, Cat. #sc-7961 |
| Cyclin D1 | Mouse, monoclonal | 1:500 | Santa Cruz Biotechnology, Cat. #sc-450 |
| c-Myc | Mouse, monoclonal | 1:1000 | Santa Cruz Biotechnology, Cat. #sc-56634 |
| p53 | Mouse, monoclonal | 1:500 | Santa Cruz Biotechnology, Cat. #sc-47698 |
| IL-4 | Rabbit, monoclonal | 1:1000 | Cell Signaling Technology, #12227 |
| IL-6 | Mouse, monoclonal | 1:500 | Santa Cruz Biotechnology, Cat. #sc-28343 |
| Bid | Mouse, monoclonal | 1:500 | Santa Cruz Biotechnology, Cat. #sc 56025 |
| Bcl2 | Mouse, monoclonal | 1:1000 | Cell Signaling Technology, #15071 |
| XIAP | Rabbit, monoclonal | 1:1000 | Cell Signaling Technology, #14334 |
| Bcl-XL | Mouse, monoclonal | 1:500 | Santa Cruz Biotechnology, Cat. #sc-56021 |
| Cyto-C | Rabbit, monoclonal | 1:1000 | Cell Signaling Technology, #4280 |
| Apaf-1 | Mouse, monoclonal | 1:500 | Santa Cruz Biotechnology, Cat. #sc-135836 |
| NF-κB | Mouse, monoclonal | 1:1000 | Cell Signaling Technology, #6956 |
| Iκκβ | Mouse, monoclonal | 1:500 | Santa Cruz Biotechnology, Cat. #sc-56918 |
| Caspase-3 | Mouse, monoclonal | 1:500 | Santa Cruz Biotechnology, Cat. #sc-56053 |
| MCL-1 | Mouse, monoclonal | 1:500 | Santa Cruz Biotechnology, Cat. #sc-53951 |
| Caspase-9 | Mouse, monoclonal | 1:500 | Santa Cruz Biotechnology, Cat. #sc-70505 |

| 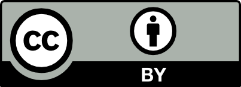 | © 2019 by the authors. Licensee MDPI, Basel, Switzerland. This article is an open access article distributed under the terms and conditions of the Creative Commons Attribution (CC BY) license (http://creativecommons.org/licenses/by/4.0/). |
| --- | --- |
